# Histone deacetylase inhibitors VPA and WT161 ameliorate the pathological features and cognitive impairments of the APP/PS1 Alzheimer’s disease mouse model by regulating the expression of APP secretases

**DOI:** 10.1186/s13195-024-01384-0

**Published:** 2024-01-20

**Authors:** Miaomiao Zhang, Wanyao Wang, Qun Ye, Yun Fu, Xuemin Li, Ke Yang, Fan Gao, An Zhou, Yonghui Wei, Shuang Tian, Shen Li, Fengjiang Wei, Wentao Shi, Wei-Dong Li

**Affiliations:** 1 Department of Genetics, School of Basic Medical Sciences, Tianjin Medical University, Tianjin, 300070 China; 2 Prenatal Diagnostic Center, Yiwu Maternity and Children Hospital, Yiwu, 322000 China; 3 College of Clinical Medicine for Obstetrics and Gynecology and Pediatrics, Fujian Maternity and Child Health Hospital, Fujian Medical University, Fuzhou, 350000 China; 4 Laboratory of Biological Psychiatry, Institute of Mental Health, Tianjin Anding Hospital, Mental Health Center of Tianjin Medical University, Tianjin, 300222 China

**Keywords:** VPA, WT161, Alzheimer’s disease, Histone deacetylase, Aβ deposition, Cognitive function

## Abstract

**Background:**

Alzheimer’s disease (AD) is a degenerative neurological disorder. Recent studies have indicated that histone deacetylases (HDACs) are among the most prominent epigenetic therapy targets and that HDAC inhibitors have therapeutic effects on AD. Here, we identified sodium valproate (VPA), a pan-HDAC inhibitor, and WT161, a novel HDAC6 selective inhibitor, as potential therapeutic agents for AD. Underlying molecular mechanisms were investigated.

**Methods:**

A cellular model, N2a-APPswe, was established via lentiviral infection, and the APPswe/PSEN1dE9 transgenic mouse model was employed in the study. LC–MS/MS was applied to quantify the concentration of WT161 in the mouse brain. Western blotting, immunohistochemical staining, thioflavin-S staining and ELISA were applied to detect protein expression in cells, tissues, or serum. RNA interference was utilized to knockdown the expression of specific genes in cells. The cognitive function of mice was assessed via the nest-building test, novel object recognition test and Morris water maze test.

**Results:**

Previous studies have focused mainly on the impact of HDAC inhibitors on histone deacetylase activity. Our study discovered that VPA and WT161 can downregulate the expression of multiple HDACs, such as HDAC1 and HDAC6, in both AD cell and mouse models. Moreover, they also affect the expression of APP and APP secretases (BACE1, PSEN1, ADAM10). RNA interference and subsequent vitamin C induction further confirmed that the expression of APP and APP secretases is indeed regulated by HDAC1 and HDAC6, with the JNK pathway being the intermediate link in this regulatory process. Through the above pathways, VPA and WT161 effectively reduced Aβ deposition in both AD cell and mouse models and significantly improved cognitive function in AD mice.

**Conclusions:**

In general, we have discovered that the HDAC6-JNK-APP secretases cascade is an important pathway for VPA and WT161 to exert their therapeutic effects on AD. Investigations into the safety and efficacy of VPA and WT161 were also conducted, providing essential preclinical evidence for assessing these two epigenetic drugs for the treatment of AD.

**Supplementary Information:**

The online version contains supplementary material available at 10.1186/s13195-024-01384-0.

## Introduction

Alzheimer’s disease (AD) is a neurodegenerative disease in which a combination of genetic and environmental factors contributes to the onset and progression of the disease [1, 2]. Senile plaques formed by amyloid-β (Aβ) are one of the main pathological hallmarks exhibited by the brain tissue of Alzheimer’s patients [3]. Aβ is a hydrolysis product of the type I transmembrane glycoprotein amyloid precursor protein (APP). Beta-secretase (β-site of APP cleaving enzyme, BACE1) [4] and the catalytic subunit of γ-secretase (presenilin 1, PS-1) [5] cleave APP to neurotoxic Aβ42, which accumulates and deposits in the brain to form senile plaques. In contrast, α-secretase (A disintegrin and metalloprotease 10, ADAM10) tends to degrade APP to the non-neurotoxic Aβ40 [6].

The *APP* gene is located on chromosome 21, and the extra copy of *APP* causes the overproduction of Aβ in Down’s syndrome patients, resulting in a young-onset AD phenotype [7]. In patients with translocation Down'’ syndrome, the presence or absence of the *APP* gene in the translocation region determines whether the patient will eventually develop an AD phenotype [8, 9]. In addition, several studies have shown that anti-Aβ monoclonal antibodies can clear Aβ plaques and somewhat slow cognitive decline in AD patients [10–14]. However, due to their high cost, monoclonal antibodies are challenging to widely employ in clinical practice. Therefore, it is important to further investigate the use of small molecules to reduce Aβ deposition in brain tissue.

Histone deacetylases (HDACs) play an important role in the regulation of chromatin remodeling and gene expression. They are also involved in several biological processes, including neurogenesis, neurodevelopment, synaptic structure and function changes and the regulation of cognitive and memory-related processes [15–17]. Overexpression of HDACs is closely associated with Aβ42 deposition and the initiation and progression of AD [18–26]. People are paying more attention to the efficacy and mechanisms of HDAC inhibitors (HDACis) in the treatment of neurodegenerative disorders [27, 28]. However, the research on HDACis for neurodegenerative diseases is still in its early stages, and more investigations are required to determine the most effective isoform(s) that can provide significant therapeutic benefits without causing serious side effects [29].

In the present study, we constructed cellular and mouse models of Alzheimer’s disease. Two HDAC inhibitors, the pan-HDAC inhibitor sodium valproate (VPA), and the novel HDAC6-specific inhibitor WT161, which is currently used for antitumor therapy [30, 31], were chosen to observe the effects of both on the expression levels of Aβ in cells and mouse brains, and on the improvement of behaviour and memory in AD mice, aiming to explore the underlying molecular mechanisms.

## Method

### Cells

Wild-type mouse neuroblastoma cells (Neuro-2a, N2a) were utilized in addition to two modified cell lines: N2a-APPswe cells, which stably express human amyloid precursor protein APP from Swedish family mutation (K595N/M596L), and N2a-APPswe cells with knockdown of HDCA1 and HDAC6, which served as model cells. The N2a cell line was acquired from Beijing Beina Genentech Co, while the overexpression lentivirus for APPswe was obtained from Shanghai Jikai Gene Medical Technology Co. Cells were cultured in high-glucose DMEM (HyClone) supplemented with 10% foetal bovine serum (FBS, Procell) and 1% penicillin/streptomycin (P/S, GIBCO) at 37 °C in a 5% CO_2_ incubator.

### Lentiviral infection and screening of stable cell lines

The cells were digested using trypsin, centrifuged and diluted to a concentration of 4 × 10^4^ cells/ml using the cell culture medium. The cells were then mixed thoroughly, seeded into 6-well plates (2 ml/well), transferred to a 37 °C incubator for 16–24 h, and randomly divided into the (1) control group, (2) negative control group and (3) transfection group when the cell fusion reached 20–30% (2 replicates per group). The control group received 1 ml of P/S-free DMEM. The negative control group received 100 μl of 1 × 10^8^ TU/ml negative virus and 900 μl of P/S-free DMEM. The infection group received 100 μl of 1 × 10^8^ TU/ml APPswe overexpression lentivirus or shHDAC lentivirus and 900 μl of P/S-free DMEM. After 12 h, the cells were changed to complete medium. The infection efficiency was observed under a microscope after 72 h. Once the cell fusion reached 70–80%, DMEM containing 3 μg/ml puromycin was added until the control cells were completely killed by puromycin (48 h). The concentration of puromycin was gradually reduced, and the infected cells were screened while collecting the cells for Western blotting to verify the expression level of the target gene. The verified cells were then frozen for seeding.

### Animals

Double-transgenic AD mice (APPswe/PSEN1dE9), known as APP/PS1 mice, were purchased from Jiangsu Collective Pharmachem Biotechnology Co. Wild-type (WT) mice on a 4-month B6C3F1 background from the same litter were used as controls. All mice were housed at the Experimental Animal Center of Tianjin Medical University in single cages of 3–5 mice in a controlled environment of 22–25 °C, 40–60% humidity, ≤ 60 dB noise barrier and 12 h light–dark cycle with free access to standard food and water. Fifty-four female APP/PS1 mice were randomly divided into the APP/PS1 model group, VPA group (50 mg/kg/day) and WT161 group (10 mg/kg/day), with 18 mice in each group. Additionally, 18 mice from the same litter were assigned as the WT control group. The mice were given intraperitoneal injections, with the WT and APP/PS1 groups receiving the same volume of saline, which were administered daily for 18 consecutive weeks. The mice were executed 8 h after the last administration of VPA, WT161 or saline, and the brain tissues were extracted and stored at − 80 °C. The dosages of VPA (50 mg/kg/day) and WT161 (10 mg/kg/day) are both determined based on published literature [30, 32]. The experiments were approved by the Institutional Animal Ethics Committee of Tianjin Medical University (TMUaMEC 2022020) and all experiments were conducted in accordance with the 3R principles and animal ethics regulations.

### WT161 brain concentration assay

The analyte stock solution was diluted to the desired working solution concentration with DMSO, and 3 μL of the working solution was added to 30 μL of wild-type B6C3F1 mouse brain homogenate to form a total volume of 33 μL of calibration standards (1, 2, 5, 10, 20, 50, 100, 500, 1000 ng/mL). Four quality control samples at concentrations of 2, 5, 50 and 800 ng/mL for brain tissues were independently prepared from those used for the calibration curves. The quality control samples were processed on the day of analysis using the same method as the calibration standards. Thirty-three microlitres of standard, QC sample and WT161 treatment group mouse brain tissue homogenate was taken, and 200 μL of IS-containing acetonitrile mixture was added for protein precipitation. The mixture was vortexed, centrifuged for 15 min at 4000 rpm at 4℃, and the supernatant was diluted three times with water. Ten microlitres of the diluted supernatant was then injected into the LC/MS system for quantitative analysis. The HPLC was performed on Shimadzu Nexera Series Pump LC-40, and the mass spectra were performed on an AB Sciex Triple Quad 5500 + LC/MS instrument.

### Mouse genotyping

Fourteen days after birth, genomic DNA was extracted from the toes of mice and used as a template for PCR amplification to identify the genotypes of WT and APPswe/PSEN1dE9 mice. Amplification was performed using primers for *APP*, *PS1* and internal reference genes. The primer sequences used were as follows: APP-f-GACTGACCACTCGACCAGGTTCTG, APP-r-CTTGTAAGTTGGATTCTCATATCCG, psen1-f-AATAGAGAACGGCAGGAGCA, Psen1-R-GCCATGAGGGCACTAATCAT, GAPDH-F-CTAGGCCACAGAATTGAAAGATCT and GAPDH-R-GTAGGTGGAAATTCTAGCATCATCC. PCR amplification parameters were set as follows: predenaturation at 94 °C for 2 min, denaturation at 94 °C for 30 s, annealing at 60 °C for 30 s, and extension at 72 °C for 40 s (35 cycles) with thorough extension at 72 °C for 5 min. The PCR products were mixed with 6 × loading buffer and loaded onto a 1% agarose gel containing GelRed dye. The gel was then electrophoresed at 120 V with 30 mM for 25 min. The separated DNA fragments were visualized under UV light using a gel imaging system, and the resulting electrophoretic images were captured and saved for further analysis.

### Quantitative real-time PCR

Total RNA was isolated from the Mouse Cerebral Cortex using TRIzol reagent (Invitrogen), followed by reverse transcription to cDNA using the HiScript lll Reverse Transcriptase kit (Vazyme). Gene expression was detected on a 7500 Real-Time PCR system (Thermo Fisher) using ChamQ Blue Universal SYBR qPCR Master Mix (Vazyme). PCR mix were prepared individually by mixing with the following sense primer and antisense primer: 5′-aggtcggtgtgaacggatttg-3′ and 5′-tgtagaccatgtagttgaggtca-3′ for a 123 base pair (bp) product of the mouse *Gapdh* gene; 5′-tcagggaccaaaacctgcat-3′ and 5′-gcaccagttctggatggtca-3′ for a 126 base pair (bp) product of the human *APP* gene; *Gapdh* expression levels were used to normalize the expression of target genes and the results were quantified using the 2^−∆∆Ct^ method. All assays were performed in quadruplicate.

### Protein sample preparation

For cellular whole proteins, the medium was removed and the cells were washed twice with ice-cold PBS before being lysed using Enhanced RIPA Lysis Buffer containing 1 mM phenyl methyl sulfonyl fluoride (PMSF, Applygen) and 1 mM complete protease inhibitor cocktail (Applygen) on ice for 20 min. The lysate was then centrifuged at 4 °C for 15 min at 12,000 × *g*, and the protein concentration of the supernatant was determined using the BCA Protein Assay Kit (Solarbio).

For tissue whole protein extraction, animal tissues were first washed with PBS to remove any blood stains, cut into small pieces and weighed in a 1.5-ml centrifuge tube. Fresh protein lysis buffer was added in 10-fold volume, and the mixture was sonicated on ice to remove any viscosity (six cycles of 15 s on, 45 s rest). The lysate was then centrifuged at 4 °C for 15 min at 12,000 × *g*, and the protein concentration of the supernatant was determined using the BCA Protein Assay Kit.

### Western blot

Equal quantities of protein samples were separated by SDS-PAGE on a 10% gel and transferred to polyvinylidene difluoride (PVDF) membranes (Millipore). The membranes were blocked in 5% (w/v) nonfat milk (Sangon Biotech) in TBST for 1 h at room temperature and incubated overnight with primary antibodies at 4 °C. The membranes were washed three times for 10 min each in TBST, incubated with the corresponding secondary antibody at room temperature for 1.5 h and washed as described above. The membranes were subjected to a chemiluminescent reaction by ECL (Life-iLab). The primary antibodies used in the study included the following: anti-β-actin (ab213262, Abcam), anti-BACE1 (A5095, Bimake), anti-ADAM10 (A5298, Bimake), anti-HDAC1 (BS6485, Bioworld), anti-HDAC2 (K107348P, Solarbio), anti-HDAC6 (ab239362, Abcam), anti-SIRT1 (#9475, Cell Signaling Technology), anti-SIRT2 (A5637, Bimake), anti-c-Jun (A5730, Bimake), anti-JNK3 (A5677, Bimake), anti-p-JNK (AP0631, Abclone) and anti-6E10 (#803014, Biolegend). The secondary antibodies included goat anti-rabbit IgG-HRP conjugate (#S0001, Affinity) and goat anti-mouse IgG-HRP conjugate (#S0002, Affinity).

### Determination of organ weight ratios and serum biochemical indexes in mice

During the animal experimentation process, the weight of the added and remaining mouse food was regularly measured and recorded each week, along with the weight of the mice. $\mathrm{The}\;\mathrm{weekly}\;\mathrm{cumulative}\;\mathrm{food}\;\mathrm{intake}\;\mathrm{per}\;10\;\mathrm{grams}\;\mathrm{of}\;\mathrm{body}\;\mathrm{weight}\;\mathrm{for}\;\mathrm{each}\;\mathrm{mouse}=\frac{Amount\;of\;added\;chow-amount\;of\;remaining\;chow}{Total\;body\;weight}$. The brains of the mice were removed and weighed, and the heart, liver, spleen, lungs, and kidneys were dissected by cutting open the abdominal and thoracic cavities and weighed. $organ\;weight\;ratios(\%)=\frac{Organ\;weight}{Body\;weight}$. The mice were fasted for 8 h 1 day before euthanasia, and their eyeballs were removed with forceps. Fresh blood was collected in EP tubes and centrifuged at 3800 rpm for 10 min after standing for 2 h at room temperature. The supernatant serum was then separated and measured according to the instructions of each kit. Blood glucose (GLU), total cholesterol (T-CHO), triglycerides (TG), high-density lipoprotein (HDL) and low-density lipoprotein (LDL) were used to evaluate cardiovascular health. Aspartate transaminase (AST), alanine transaminase (ALT) and total bilirubin (T-BIL) were used to evaluate liver health, while creatinine (Cr) and blood urea nitrogen (BUN) were used to evaluate kidney health.

### Perfusion and brain tissue extraction

Mice were deeply anaesthetized by intraperitoneal injection of 5 ml/kg 20% urethane. Mice were considered fully anaesthetized when they were lying supine with even heartbeat and breathing, relaxed muscles, no whisker touch response and no limb movements. After confirming complete anaesthesia, the limbs were fixed, and the abdominal and thoracic cavities were opened to expose the heart and liver. A perfusion needle was inserted into the left ventricle and fixed in position, and the inferior vena cava was cut open to allow venous blood to flow out. Rapidly perfuse 1–3 ml physiological saline until the liver and heart turn white and the outflowing blood becomes clarified. Then, the mice were slowly perfused with 4% precooled paraformaldehyde (PFA) and fixed for 15–20 min. The infusion was considered successful when the mouse’s tail became stiff and straight, the limbs became rigid, and organs with abundant blood flow, such as the liver, spleen and kidneys, turned grayish-white and the mouse’s ear tips, lips and paw pads also turned pale. Then, the posterior neck muscles were carefully removed, and the skull was carefully dissected with forceps to remove the brain. The surface meninges were peeled off, and the optic nerves were cut. The perfused brain tissue was white and firm with no visible red blood vessels. Then, the cells were fixed in a fixative solution for 12 h.

### Paraffin section and immunohistochemical staining

After the tissues were fixed, they were dehydrated in graded ethanol solutions (70, 95 and 100%) for 30 min and cleared in xylene for 30 min to transparent. The samples were infiltrated with molten paraffin wax at 60 °C for 2–3 h, embedded using a LEICA EG1150 paraffin embedding machine, and then cut into 4–5-μm sections using a LEICA RM 2255 paraffin rotary microtome. The sections were dried at room temperature and dried in an oven at 60 °C to remove remaining moisture. To perform thioflavin-S fluorescence staining (MedChemExpress, cat# HY-D0972), paraffin-embedded sections were deparaffinized in xylene and rehydrated through a graded alcohol series. The sections were then stained with 0.0125% toluidine blue solution at room temperature for 5–10 min, followed by incubation in 5 × PBS buffer at 4 °C. The preheated paraffin sections were subjected to xylene dewaxing and rehydrated in a gradient alcohol series. Drops of 3% H_2_O_2_ in methanol were added to the sections, incubated at room temperature for 5–10 min, then soaked in TBS buffer solution and washed twice for 3 min each time. Antigen retrieval was performed by heating the Tris–EDTA solution to boiling in a microwave oven at high heat, and the washed sections were added and soaked for 10–20 min. After washing, the tissue was covered with 5% BSA and incubated at room temperature for 30 min. The diluted primary antibody was then added to the tissue and incubated overnight in a wet box at 4 °C, followed by a 20-min rewarming period in the wet box. After washing, an appropriate amount of the diluted working solution of the secondary antibody (HRP labeled with horseradish peroxidase) was added to the tissue and incubated at room temperature for 1 h. DAB staining solution was added and allowed to stand for 5–10 min for staining. The nucleus was slightly retained with haematoxylin dye, observed under the microscope and terminated in time. The residual dye was washed away, and the sections were dehydrated in a gradient alcohol series and cleared in xylene. Finally, neutral gum was added to the slides to seal the coverslip.

### ELISA

Sandwich-enzyme-linked immunosorbent assay (sandwich-ELISA) was used to detect the content of Aβ42 (Elabscience). Aβ42 in the sample or standard substance was bound to Aβ42 antibody coated on the solid phase carrier of an enzyme-labeled plate. Biotin anti-Aβ42 antibody was then bound to the Aβ42 antibody, and labeled biotin was specifically bound to horseradish peroxidase (HRP)-labeled avidin to form an immune complex. The colour-developing substrate tetramethylbenzidine (TMB) was then catalysed to form a blue complex, which turned yellow after the reaction was terminated with a termination liquid. The intensity of the colour of the complex was proportional to the amount of Aβ42 in the sample.

### Novel object recognition test

A novel object recognition test was conducted to assess the learning and memory abilities of mice with an instinct to explore new objects. The experimental setup comprised a rectangular box and three objects (A, B, C), in which A was identical to B, while C was clearly different from A and B. Mice were transferred to the experimental room 24 h before the test to acclimate to the environment, and any abnormal mice were excluded from the study. On the first day of the experiment, mice were allowed to explore the behaviour box freely for 5 min. After the exploration, the behaviour box was carefully wiped with 75% alcohol to eliminate any residual odour. On the second day, A and B were fixed at the left and right ends of the sidewall of the behaviour box (10 cm away from the wall). Mice were placed in the behaviour box with the two objects facing backwards for free exploration for 5 min during the same period. After a 3-h interval, B was replaced with C, while A remained unchanged. Mice were then placed back into the behaviour box for 5 min of exploration. The automatic image acquisition system recorded the number of times mice touched objects A and C with their mouth or nose tip, as well as the exploration time when the mouse’s nose or mouth approached objects A and C within 2–3 cm. Exploratory behaviours included the mouse’s front paw on the object, sniffing, or licking the object, while resting on the stomach or lying around the object did not count as exploration time. The number and time spent exploring new and old objects were used to assess the cognitive status of the mice, with normal cognitive ability resulting in an increased tendency to explore new objects. The discrimination index (DI) was calculated as follows: DI = (New object exploration time − old object exploration time)/(New object exploration time + Old object exploration time) × 100%

### Nest-building test

Experimental mice were housed in standard resin rat boxes measuring 28 × 12 × 16 cm, with a layer of wood shavings approximately 1 cm thick added to the bottom of the cage. The mice were acclimatized to the cages for 24 h before the start of the experiment. The first day of the test began 2 h before the night rhythm. Each cage was placed in the same position, and the free movement of the mice was observed for 2 h. The latency, defined as the time when the mice first began to bite and touch the tissue paper, was recorded. If the mice did not start nesting within 2 h, the latency was recorded as 120 min. The nesting conditions of the mice were assessed blindly at 2, 24 and 48 h after the start of the experiment. The scoring criteria were based on the cohesiveness and three-dimensional structure of the nests, as follows: (1) 0 points: no tissue was touched, and no bite marks were present; (2) 1 point: tissues scattered around the cage, but no obvious signs of biting; (3) 2 points: paper towels concentrated on one side of the cage, loose and without an obvious shape of the nest, but no obvious bite marks; (4) 3 points: paper towels concentrated on one side or corner of the cage, with a small part bitten to form a visible shallow and flat nest; (5) 4 points: tissues mostly bitten and gathered into a nest, with a three-dimensional structure and integrity.

### Morris water maze test

Before the experiment, the mice were isolated in cages and brought into the test chamber. The Morris water maze (MWM) test was conducted using a round jar with a diameter of 140 cm and a height of 50 cm (Shanghai Mobil Datong Technology Co., Ltd.). The water temperature was maintained at 22–25 °C to prevent the mice from floating. An underwater escape platform measuring 10 × 10 cm was placed 1.5 cm below the milky water surface in one of the quadrants. Spatial cues of different geometric shapes were placed around the edge of the pool to aid the mice in identifying the platform location. The mice were given 1 day of solitary acclimation before beginning the acquisition training phase. Acquisition training was conducted for five consecutive days, with each mouse being tested four times per day. The experiment was considered complete if the mouse found the platform or if 60 s had elapsed. If a mouse was unable to locate the underwater platform during a given trial, it was guided to the platform. The delays and paths taken by the mice to reach the platform were tracked and recorded. Swimming speed was measured to account for motor function as a potential confounding factor. On the sixth day, a single probe test was conducted 24 h after the final trial of the acquisition phase to assess the integrity and strength of spatial memory. The outcome of the probe test was determined by analysing the amount of time spent by APP/PS1 and WT mice in the given quadrant and the average proximity to the escape platform.

### Statistical methods

The experimental data were statistically analysed using SPSS 22.0, and the experimental data results were expressed as the mean ± standard deviation ($\overline{x }$ ± s). One-way ANOVA was used to compare the mean values between multiple samples, and the Bonferroni method was used to correct the *P* value for the comparison of data between multiple groups, with *P* < 0.05 indicating significant differences. GraphPad Prism 6, Adobe Photoshop CS5, and Adobe Illustrator 2019 were used to illustrate the experimental results.

## Results

### VPA and WT161 can affect the expression levels of multiple HDACs in the AD cell model (N2a-APPswe)

**Fig. 1:**
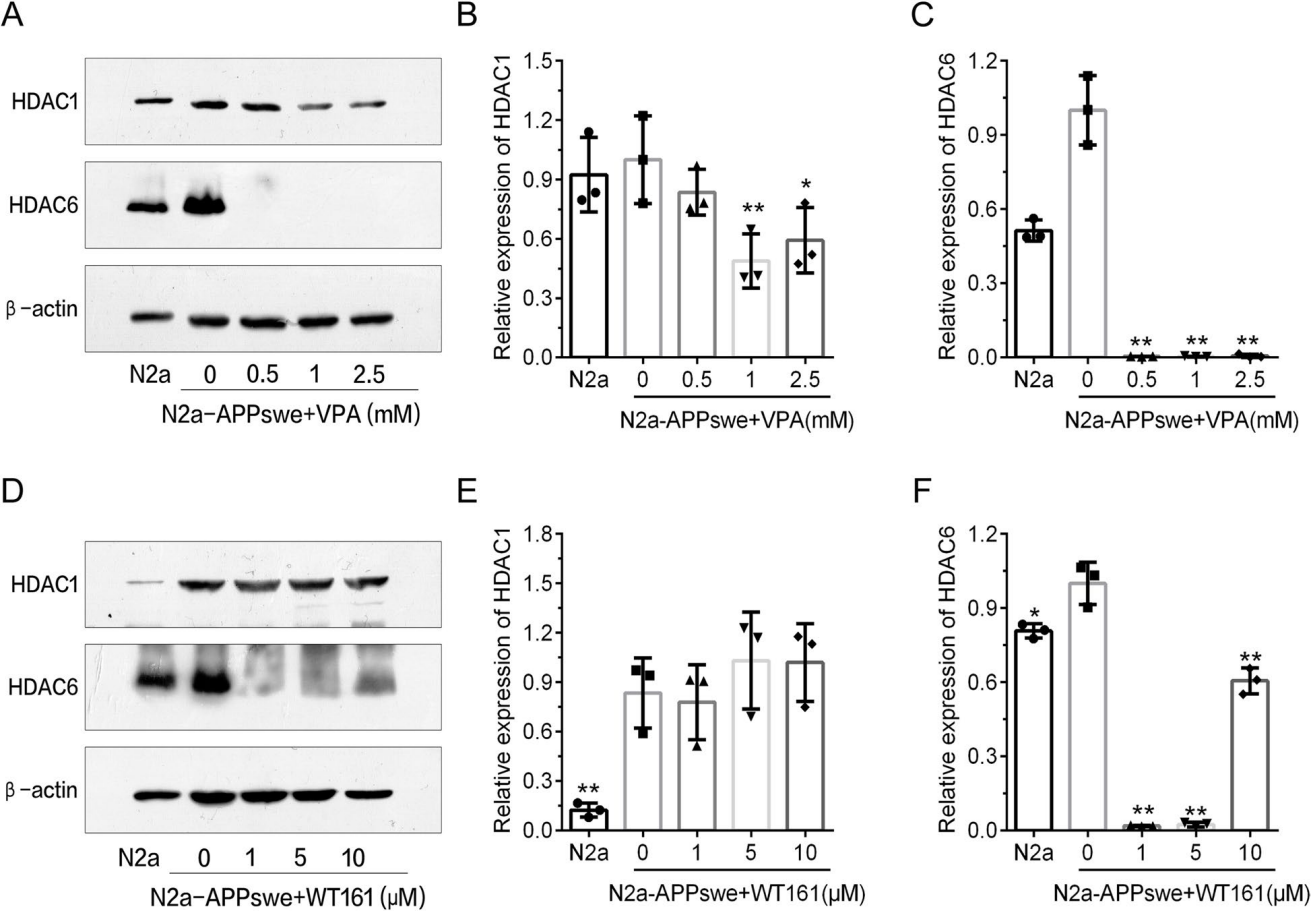
Effect of VPA and WT161 on the expression of HDAC1 and HDAC6 in N2a-APPswe. **A** Western blot detection of HDAC1 and HDAC6 expression in N2a-APPswe treated with different concentration gradients of VPA for 72 h. **D** Western blot detection of HDAC1 and HDAC6 expression in N2a-APPswe treated with different concentrations of WT161 for 72 h. **B**, **C**, **E**, **F** The results of greyscale scan analysis ($\overline{x }$±s, *n* = 3), in which N2a-APPswe treated with 0 μM VPA and WT161 were used as the baseline for one-way ANOVA to compare the differences with other treatment groups, *P < 0.05 and **P < 0.01

We established the appropriate concentration gradients for VPA and WT161 (VPA: 0.5/1.0/2.5 mM; WT161: 1.0/5.0/10.0 mM) based on the results of the cytotoxicity assay (Additional file 1: Fig. S1) to investigate the impact of both small molecules on the expression levels of several HDACs in N2a-APPswe. HDAC6 expression levels were significantly higher in N2a-APPswe than in the wild-type N2a cell line, while HDAC1 expression was not significantly changed (Fig. 1). The expression of HDAC1 and HDAC6 can be dose-dependently downregulated by VPA. WT161 reduced the expression level of HDAC6 in N2a-APPswe cells but did not affect the expression of HDAC1 (Fig. 1). The impact of VPA and WT161 on the expression of three additional histone deacetylases (SIRT1, SIRT2 and HDAC2) is shown in Additional file 1: Fig. S2.

### The proteolytic cleavage of APP in N2a-APPswe is related to the expression of HDAC1 and HDAC6

**Fig. 2:**
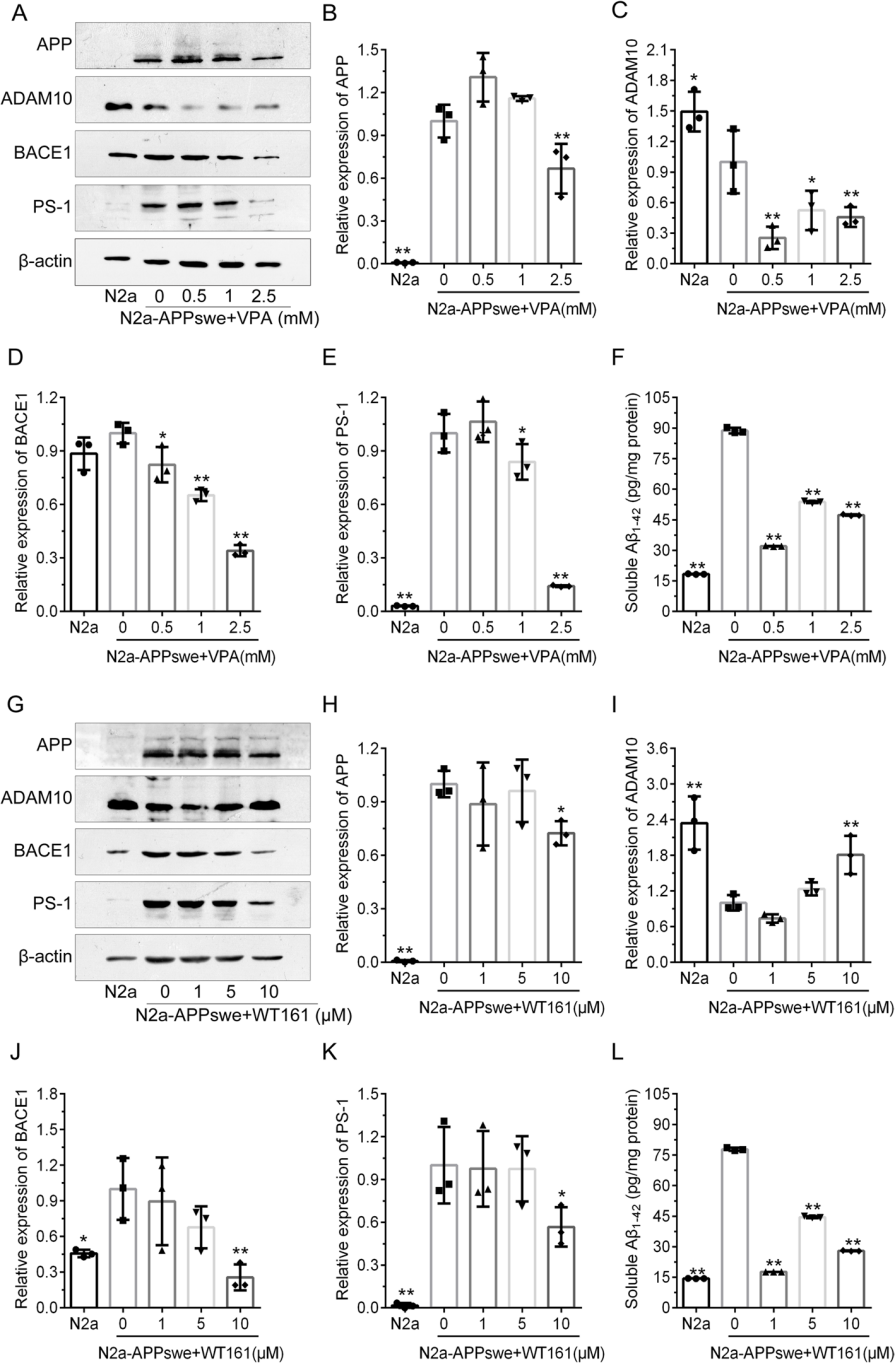
Effect of VPA and WT161 on APP metabolism-related protein expression in N2a-APPswe cells. **A** Western blot detection of APP, ADAM10, BACE1 and PS-1 expression in N2a-APPswe treated with different concentrations of VPA for 72 h. **G** Western blot detection of APP, ADAM10, BACE1 and PS-1 expression in N2a-APPswe treated with different concentrations of WT161 for 72 h. **B**–**E**, **H**–**K** The results of grayscale scan analysis ($\overline{x }$±s, *n* = 3), in which N2a-APPswe treated with VPA and WT161 in group 0 were used as the baseline, using one-way ANOVA to compare the differences with other treatment groups, *P < 0.05, **P < 0.01. **F**–**L** The soluble Aβ42 protein concentrations (detected by ELISA) in N2a-APPswe treated with different concentration gradients of VPA versus WT161 for 72 h ($\overline{x }$±s, *n* = 3), in which group 0 was used as the baseline, using one-way ANOVA to compare the differences with other treatment groups, *P < 0.05, ** P < 0.01

Expression of APP was elevated in N2a-APPswe cells. Meanwhile, BACE1 (β-secretase) and PSEN1 (γ-secretase) expression was elevated, while the expression of ADAM10 (α-secretase) was reduced, leading to a significant increase in Aβ42 concentration (Fig. 2). In a dose-dependent manner, VPA decreased the expression of APP, BACE1, PS-1 and ADAM10 and the levels of soluble Aβ42 in AD cells. On the other hand, 10 μM WT161 in the cell culture medium reduced the expression of APP, BACE1 and PS-1 and partially restored the expression of ADAM10, thereby reducing the concentration of soluble Aβ42 in N2a-APPswe (Fig. 2). In other words, VPA and WT161 showed the same trend in regulating APP, BACE1, and PS-1 expression and the opposite trend in regulating ADAM10 expression, but both could reduce the concentration of soluble Aβ42 in AD cell models.

**Fig. 3:**
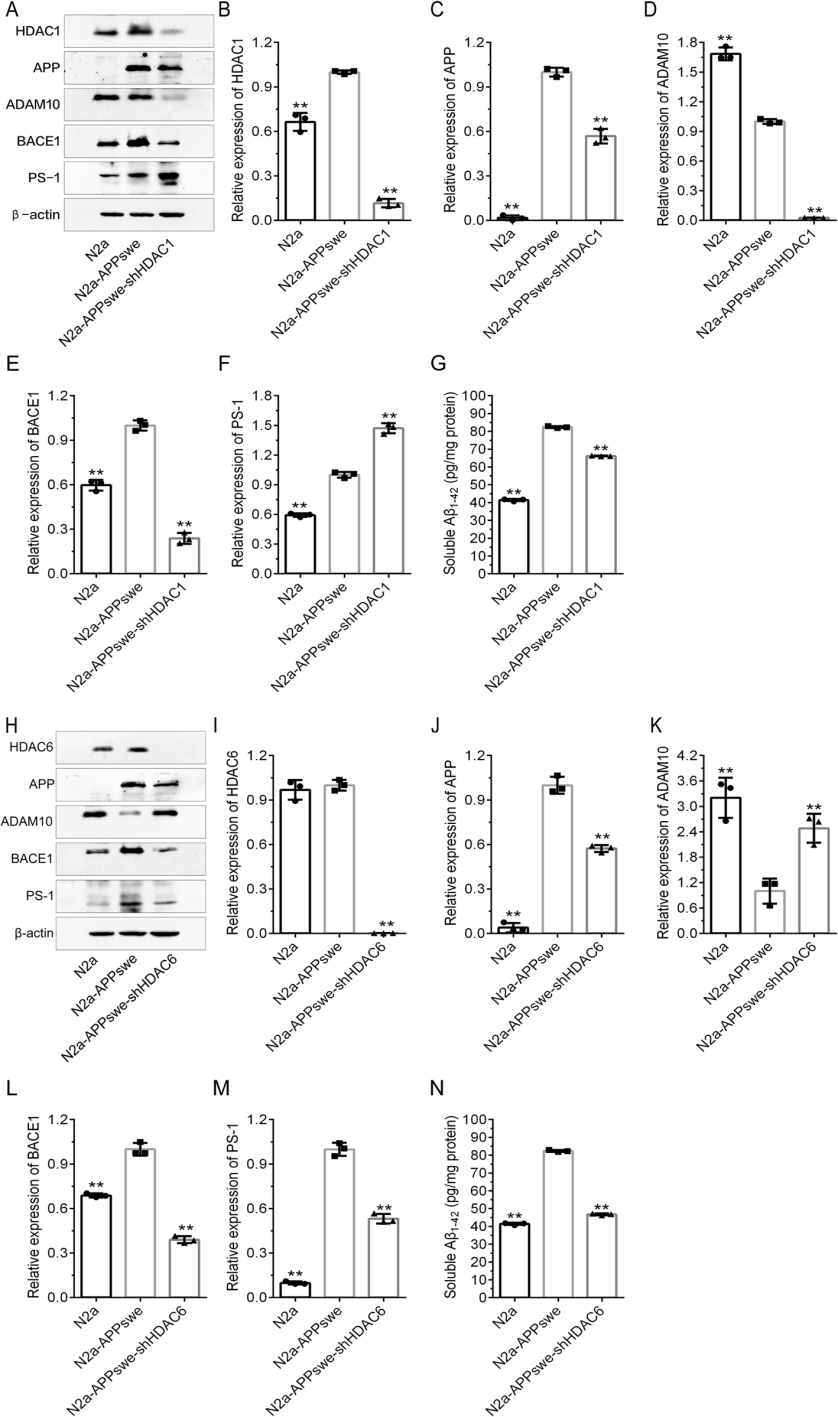
Effect of HDACs knockdown on APP metabolism-related protein expression in N2a-APPswe. **a** Western blot detection of HDAC1, APP, ADAM10, BACE1 and PS-1 expression in N2a, N2a-APPswe and N2a-APPswe-shHDAC1 cells. **b**–**f** The results of grayscale scan analysis ($\overline{x }$±s, *n* = 3), in which N2a-APPswe were treated with one-way ANOVA to compare the differences with other treatment groups, *P < 0.05, **P < 0.01. **g** Soluble Aβ42 protein concentrations in N2a, N2a-APPswe and N2a-APPswe-shHDAC1 ($\overline{x }$±s, *n* = 3). **h** Western blot detection of HDAC6, APP, ADAM10, BACE1 and PS-1 expression in N2a, N2a-APPswe and N2a-APPswe-shHDAC6 cells. **i**–**n** The results of grayscale scan analysis ($\overline{x }$±s, *n* = 3), in which N2a-APPswe was treated as the baseline, and one-way ANOVA was used to compare the differences with other treatment groups, *P < 0.05, **P < 0.01. **g** N2a, N2a-APPswe and N2a-APPswe-shHDAC6 cell soluble Aβ42 protein concentrations ($\overline{x }$±s, *n* = 3)

Then, we established the N2a-APPswe-shHDAC1 and N2a-APPswe-shHDAC6 cell lines to stably knockdown HDAC1 and HDAC6 expression in N2a-APPswe and found that APP, ADAM10 and BACE1 were positively correlated with HDAC1 expression, while PS-1 was negatively correlated with HDAC1 expression (Fig. 3). On the other hand, APP, BACE1 and PS-1 were also positively correlated with HDAC6 expression, whereas ADAM10 was negatively correlated with HDAC6 expression (Fig. 3).

Previous studies have shown that vitamin C promotes the expression of HDAC1 and HDAC6 in cultured cells [33], and the same effect was shown in the two knockdown cell lines mentioned above. Additionally, we showed that partial restoration of HDAC6 expression decreased ADAM10 expression and increased BACE-1 and PS-1 expression, while partial restoration of HDAC1 expression increased APP expression and decreased PS-1 expression (Additional file 1: Fig. S3).

### Regulation of APP secretase expression by HDAC1 and HDAC6 is dependent on the JNK pathway

**Fig. 4:**
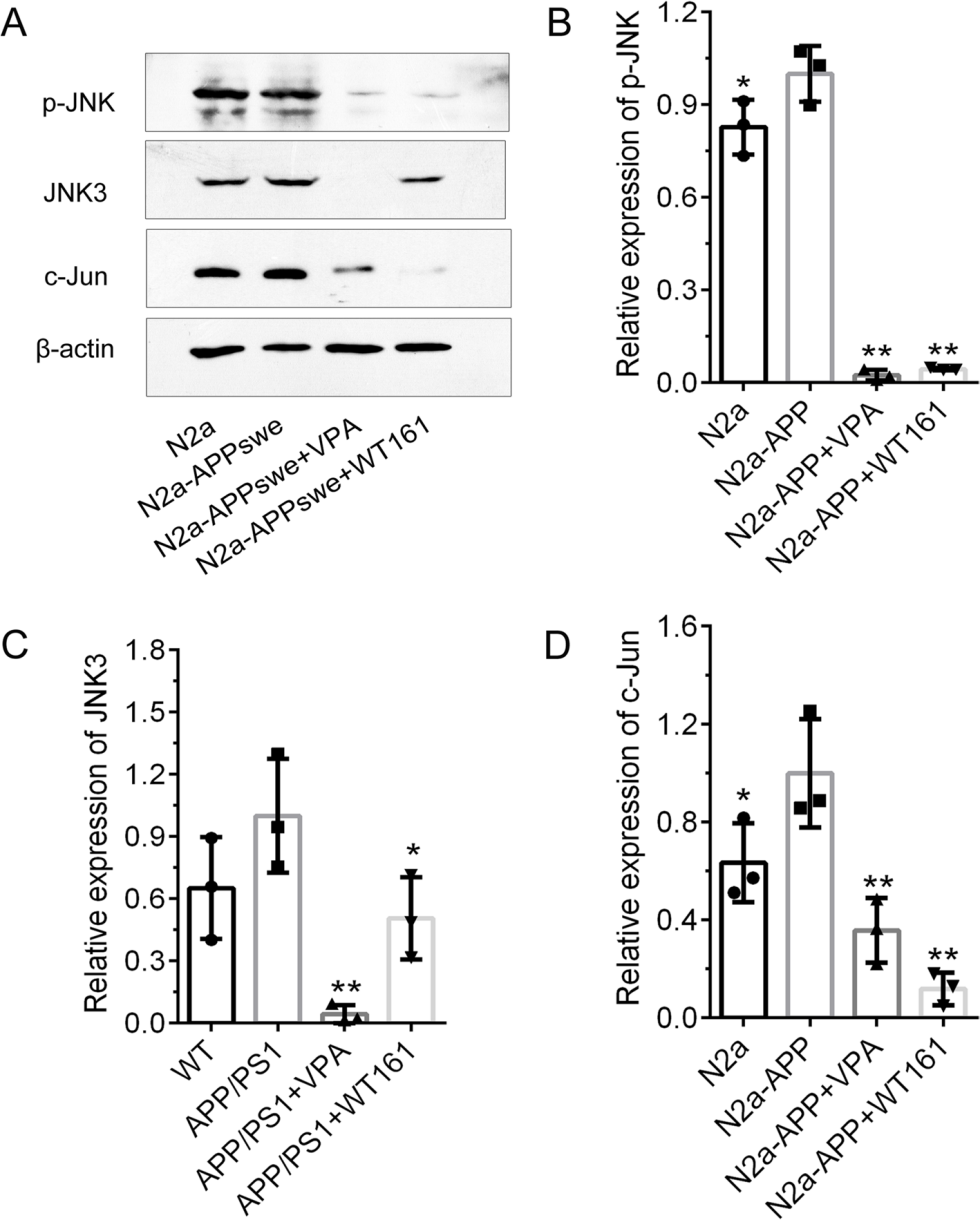
Effect of VPA and WT161 on the JNK/c-Jun pathway in N2a-APPswe. **A** Western blot detection of p-JNK, JNK3 and c-Jun expression in N2a-APPswe cells after VPA (2.5 mM) and WT161 (10 μM) treatment for 72 h. **B**–**D** The results of greyscale scan analysis ($\overline{x }$±s, *n* = 3), in which the N2a-APPswe cell group was used as the baseline and compared with other treatment groups using one-way ANOVA, *P < 0.05 and **P < 0.01

Furthermore, we noticed that the JNK inhibitor (SP600125) dose-dependently upregulated ADAM10 expression and downregulated BACE1 and PS-1 expression in N2a-APPswe (Additional file 1: Fig. S4), which is consistent with previous research demonstrating that the JNK pathway is directly involved in the regulation of APP secretase expression [34–38]. VPA and WT161 inhibited JNK phosphorylation and downregulated JNK3 and c-Jun expression levels (Fig. 4), and HDAC1 and HDAC6 were involved in this regulation (Additional file 1: Fig. S5).

According to the aforementioned findings, VPA inhibits the expression of HDAC1 and HDAC6 in the N2a-APPswe cell model, whereas WT161 specifically inhibits HDAC6 expression. HDAC1 and HDAC6 additionally influence APP expression and the expression of several APP secretases through the JNK pathway, which reduces Aβ42 accumulation in N2a-APPswe. Next, we wanted to further observe whether VPA and WT161 could improve cognitive impairment in AD mouse models and explore the molecular mechanisms by which both act in brain tissue.

### VPA and WT161 improve daily behaviour, short-term memory and spatial memory in the APP/PSEN1 transgenic mouse model of AD

We assessed the toxic effects of VPA and WT161 on mice and found that intraperitoneal injection of 50 mg/kg/day VPA and 10 mg/kg/day WT161 did not affect feeding and body weight (Additional file 1: Fig. S6) and had no effect on relative organ weight (Additional file 1: Fig. S7) but slightly affected liver function (Table S1). After 18 weeks of intraperitoneal injection, we applied LC–MS/MS to detect the concentration of WT161 in the brain of 5 mice (Table 1). The average concentration was 6.05 ng/g (approximately 0.13 μM), confirming for the first time that WT161 can penetrate the blood–brain barrier of the APP/PS1 mice.

**Table 1:** The concentration of WT161 in the brain of APP/PS1 mice

Mouse ID	Concentration (ng/g)	Mean ± SD (ng/g)
M59	5.65	6.05 ± 1.74
M137	8.95	
M144	5.75	
M150	4.25	
M152	5.65	

**Fig. 5:**
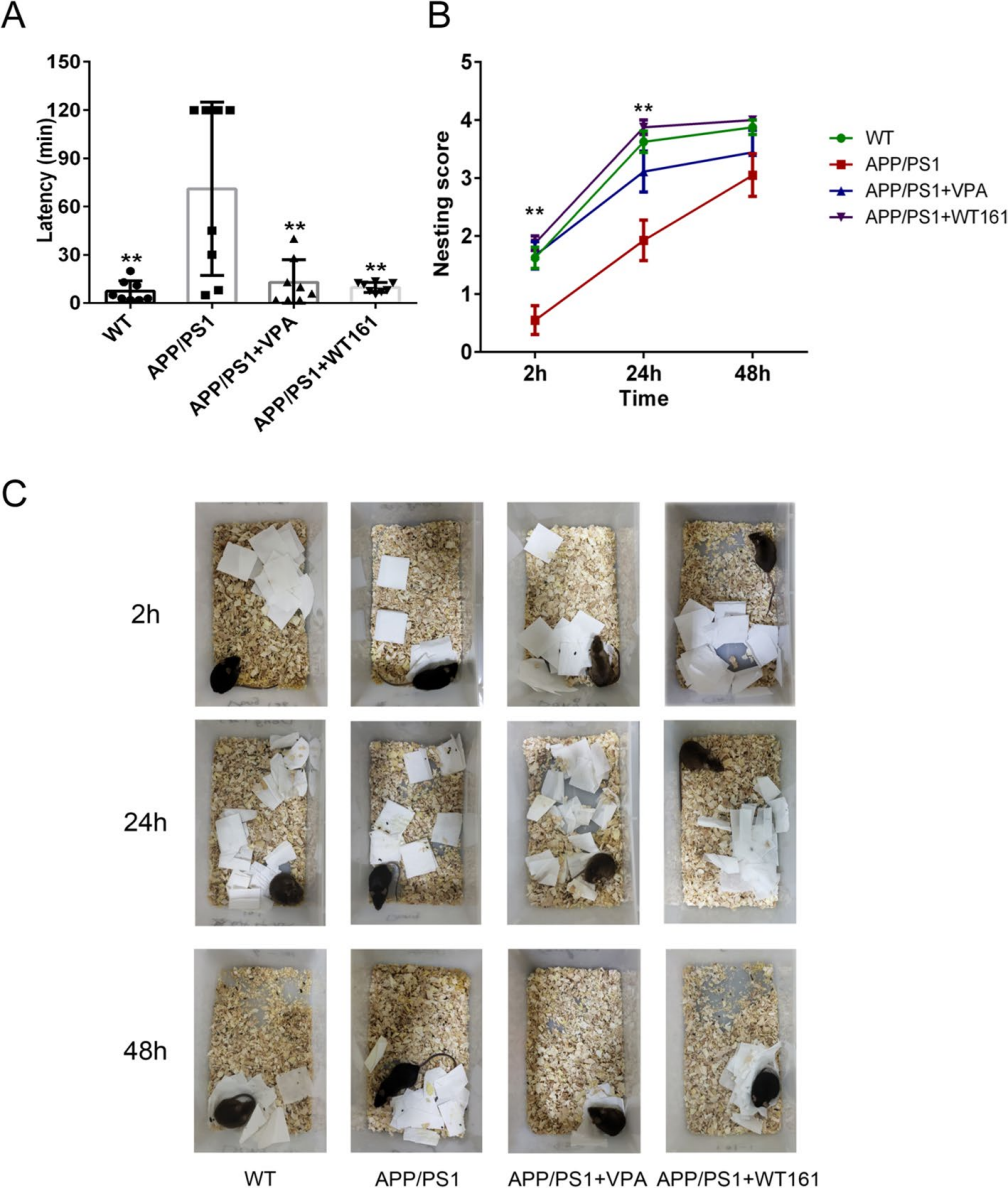
Nest-building test detects the effect on social activity and daily behavioural ability. **A** The differences between the APP/PS1 group and the other treatment groups in social activity and daily behavioural ability were compared using one-way ANOVA during the nesting latency period ($\overline{x }$±s, *n* = 8). **B** Nesting scores of mice in each group at 2, 24 and 48 h after the start of nesting, using two-way repeated-measures ANOVA to compare the effects of different treatments and times on nesting scores of mice. **C** Schematic diagram of nesting in each group at 24 h and 48 h after the start of nesting. *P < 0.05, **P < 0.01

The behavioural deficiency of APP/PSEN1 AD mice was evaluated using the nest-building test. The nesting latency of AD mice was significantly prolonged, whereas both VPA and WT161 reduced the nesting latency of AD mice to the level of wild-type mice. In addition, the nesting score of AD mice was significantly lower than that of wild-type mice at both the 2- and 24-h time points, while both VPA and WT161 restored the nest-building ability of AD mice (Fig. 5).

**Fig. 6:**
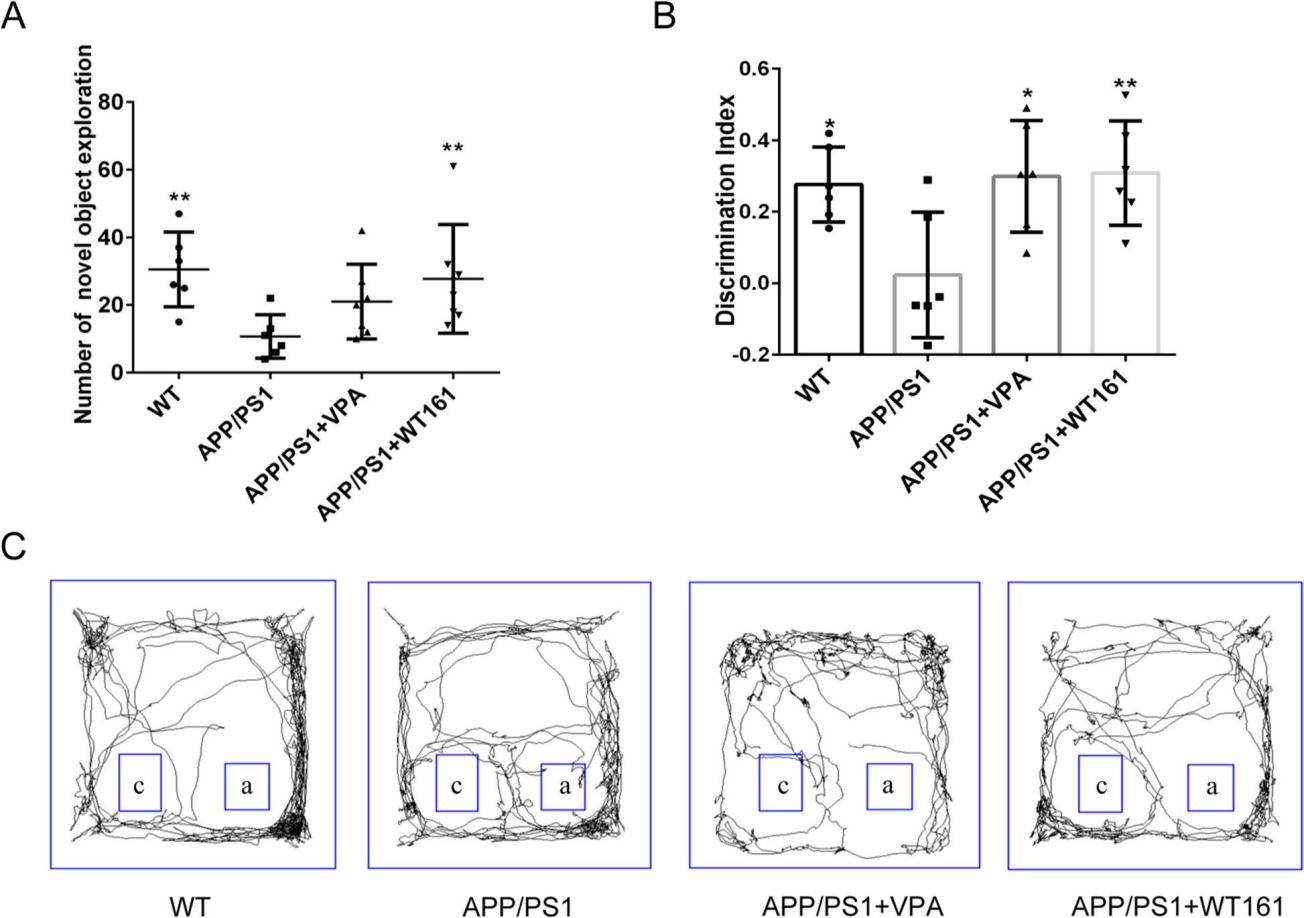
New object recognition test detecting the effect on short-term learning memory behaviour. **A** The number of times mice explored new objects, using the APP/PS1 group as the baseline, and one-way ANOVA was used to compare the differences with other treatment groups on short-term learning memory behaviour ($\overline{x }$±s, *n* = 6). **B** The discrimination index of mice in each group, using the APP/PS1 group as the baseline and comparing with other treatment groups using one-way ANOVA. **C** Trajectory pattern plots of each group of mice exploring old and new things, *a*: old things, *c*: new things. *P < 0.05 and ** P < 0.01

The learning abilities and short-term memory of the mice were evaluated using the novel object recognition test. AD mice explored new objects significantly less frequently than wild-type mice, while both VPA and WT161 significantly improved learning and short-term memory in AD mice (Fig. 6).

**Fig. 7:**
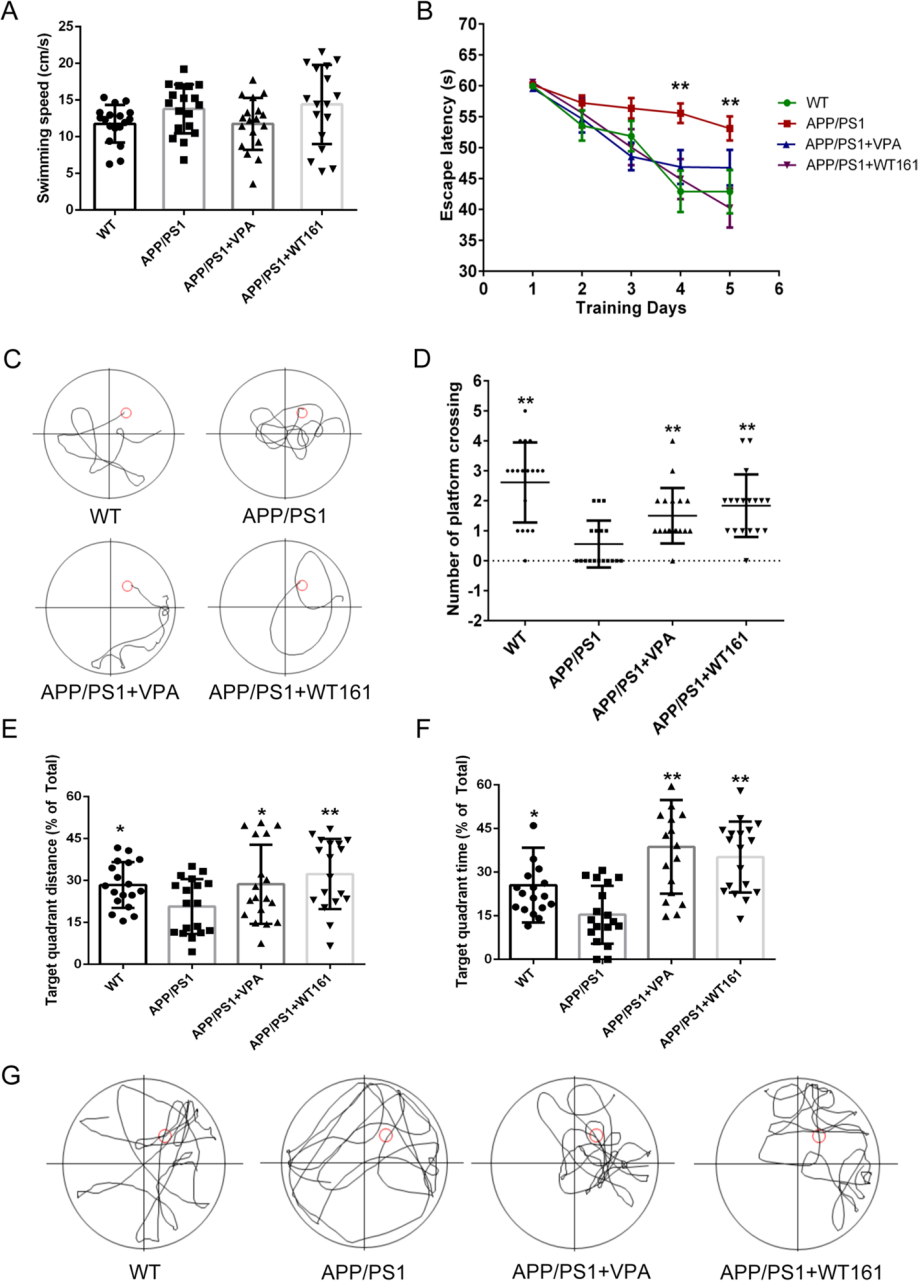
Morris water maze test detecting the effects on spatial localization and long-term memory ability. **A** Mouse swimming speed, using the APP/PS1 group as the baseline and comparing the differences with other treatment groups using one-way ANOVA ($\overline{x }$±s, *n* = 18). **b** The evasion latency of each group in the localization navigation experiment and the effect of different treatments and times on the evasion latency of mice were compared using two-way repeated-measures ANOVA. **C** Schematic diagram of the original swimming trajectory of each group of mice on the fifth day of the positioning navigation experiment. **D**–**F** denote the number of times mice crossed the original platform, the distance/total distance of the original platform quadrant and the time/total time of the original platform quadrant for each group of the spatial exploration experiment, using the APP/PS1 group as the baseline and comparing the differences with other treatment groups using one-way ANOVA. **G** Schematic diagram of the original swimming trajectory of each group of mice in the spatial exploration experiment. *P < 0.05, **P < 0.01

We used the Morris water maze test to assess the spatial memory ability of mice. The submerged escape platform was placed in the northeast quadrant of the circular swimming arena. As shown in Fig. 7A, there was no significant difference between the swimming speeds of the four groups of mice. In the hidden platform experiments on days 4 and 5, AD mice had significantly longer escape latencies than wild-type mice, whereas VPA and WT161 significantly shortened the escape latencies of AD mice (Fig. 7B). A difference can also be visualized from the mice’s initial swimming trajectories (day 5, Fig. 7C). After the hidden platform was removed, spatial probe trials were conducted. VPA and WT161 significantly increased the number of times AD mice crossed the hidden platform (Fig. 7D), increased the distance travelled (Fig. 7E), lengthened the amount of time that mice swam in the northeast quadrant (Fig. 7F), and altered the swimming trajectories of the mice (Fig. 7G).

### VPA and WT161 reduce Aβ42 deposition in the brains of AD mice via the HDAC1/HDAC6-JNK-APP secretase pathway

**Fig. 8:**
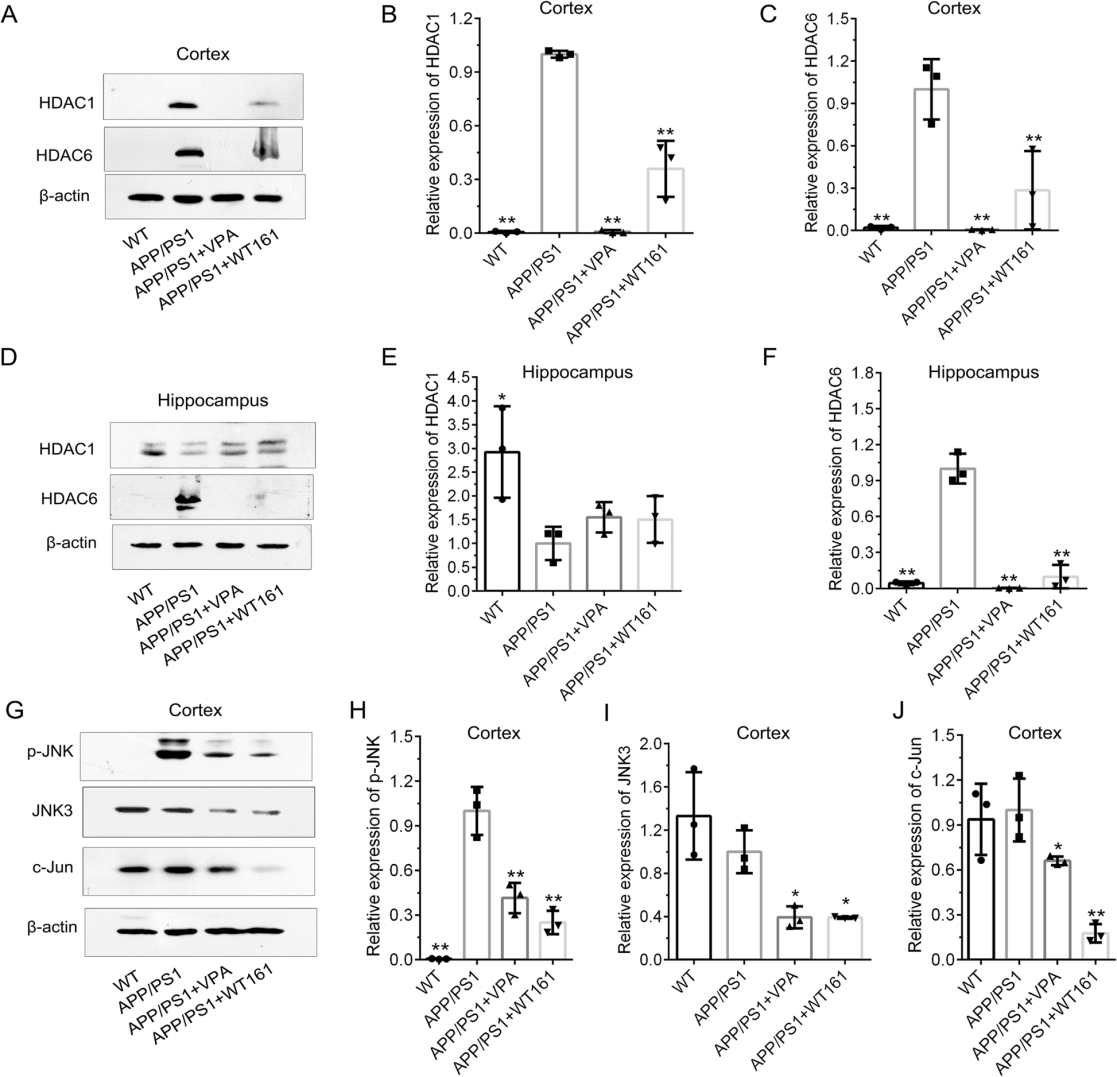
Effects of VPA and WT161 on HDACs and the JNK/c-Jun pathway. **A** Western blot detection of HDAC1 and HDAC6 expression in the mouse cortex in each group. **D** Western blot detection of HDAC1 and HDAC6 expression in the mouse hippocampus in each group. **B**, **C**, **E**, **F** The results of grayscale scan analysis ($\overline{x }$±s, *n* = 3), in which the APP/PS1 group was used as the baseline, and one-way ANOVA was used to compare the differences with other treatment groups, *P < 0.05, **P < 0.01. **G** Western blot to detect the effect of VPA and WT161 treatment on the expression of p-JNK, JNK3 and c-Jun in the cerebral cortex of APP/PS1 double transgenic AD mice. **H**–**J** The results of grayscale scan analysis ($\overline{x }$±s, *n* = 3), in which the APP/PS1 group was the baseline and compared with other treatment groups using one-way ANOVA (*P < 0.05 and **P < 0.01)

**Fig. 9:**
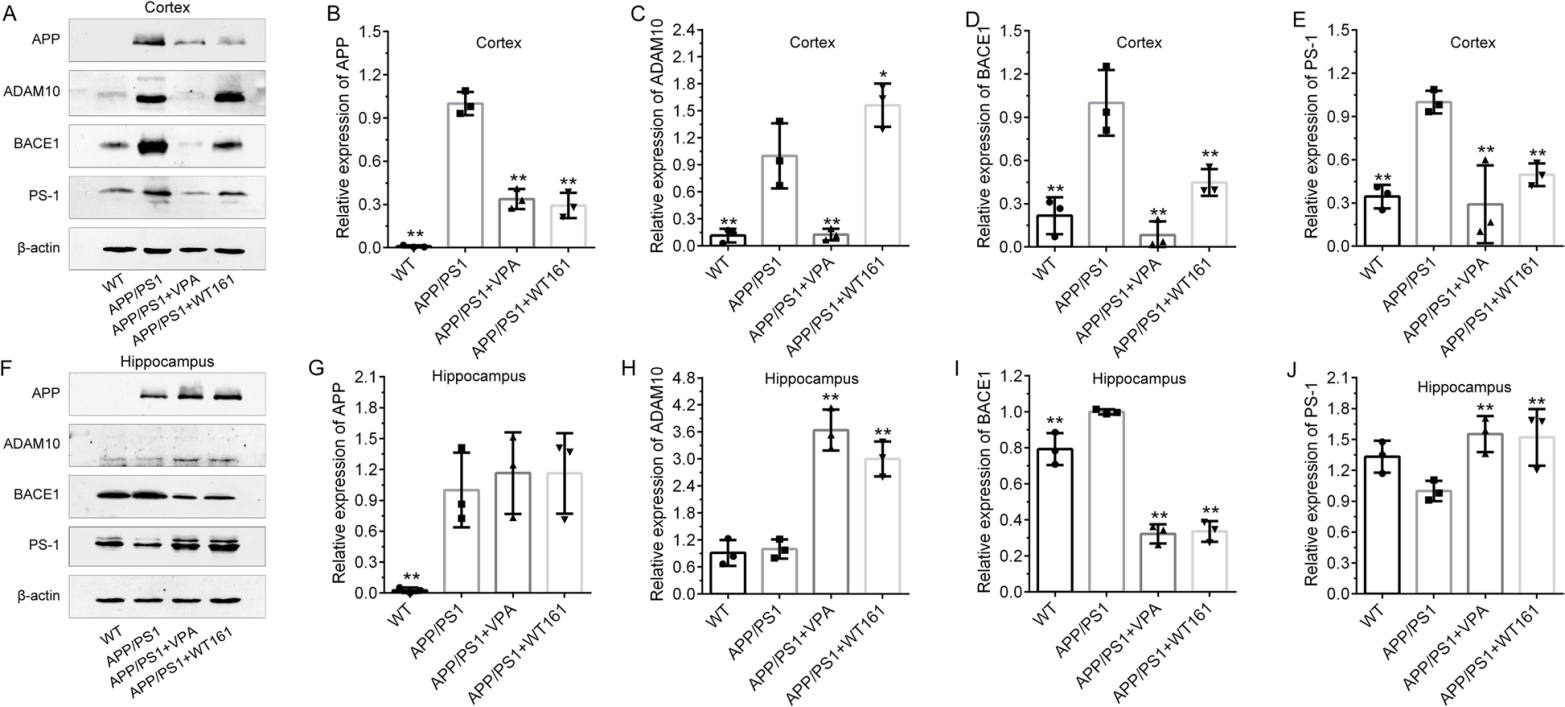
Effects of VPA and WT161 treatment on APP metabolism-related protein expression. **A** Western blot detection of APP, ADAM10, BACE1 and PS-1 expression in the cerebral cortex of each group of mice. **F** Western blot detection of APP, ADAM10, BACE1 and PS-1 expression in the hippocampus of each group of mice. **B**–**E**, **G**–**L** The results of grayscale scan analysis ($\overline{x }$±s, *n* = 3), in which the APP/PS1 group was the baseline, and compared with other treatment groups using one-way ANOVA, *P < 0.05, **P < 0.01

**Fig. 10:**
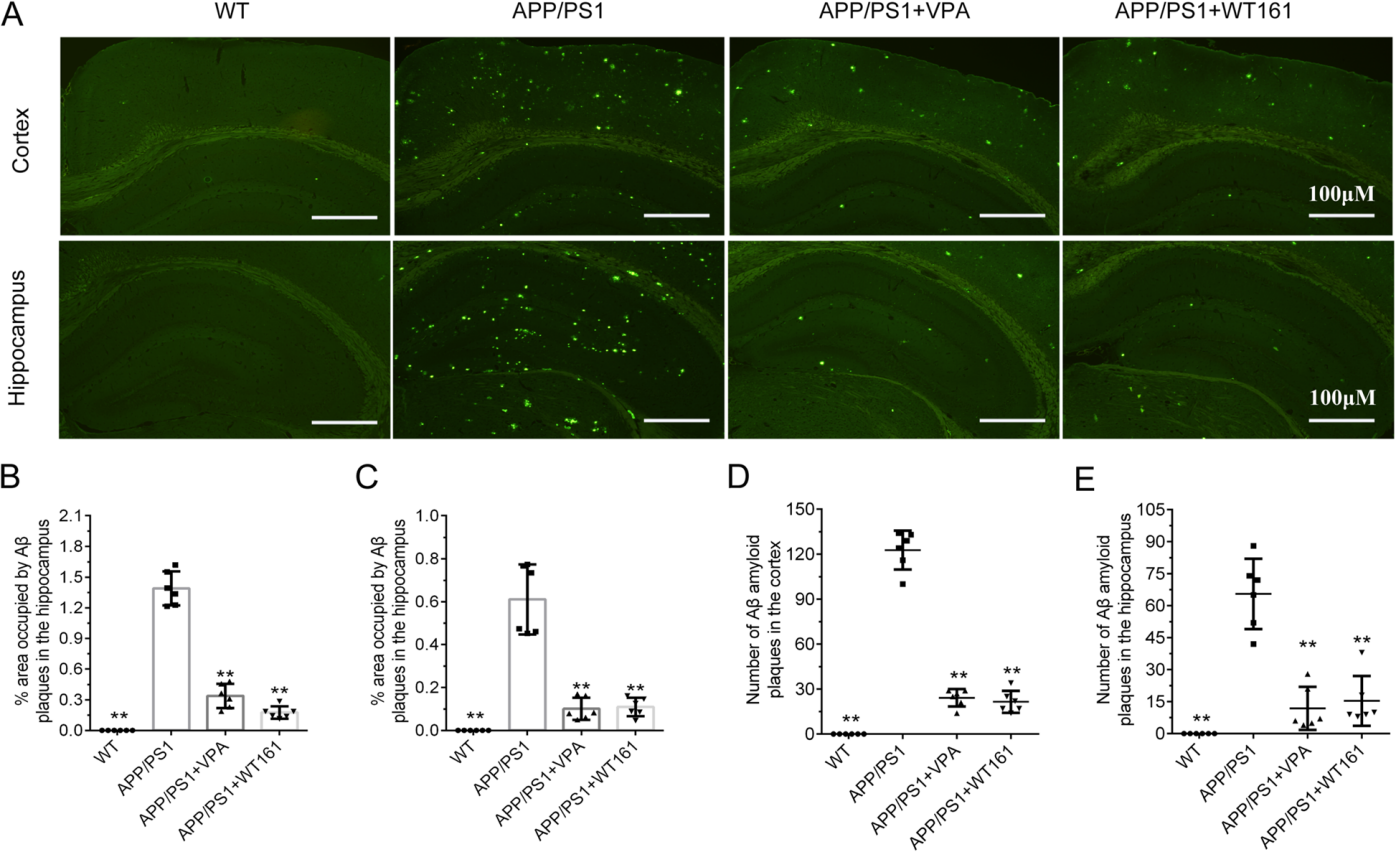
Detection of brain Aβ amyloid deposition in VPA- and WT161-treated AD mice by thioflavin-S. **A** Thioflavin-S staining (× 200, coronal cut) to detect Aβ amyloid deposition in the cortex and hippocampus of each group of mice. **B**, **C** The area ratio of Aβ amyloid plaques in the cortex and hippocampus of each group of mice ($\overline{x }$± s, *n* = 6). **D**, **E** The number of Aβ amyloid plaques in the cortex and hippocampus of each group ($\overline{x }$± s, *n* = 6), all of the above were based on the APP/PS1 group, in which the APP/PS1 group was the baseline, and compared with other treatment groups using one-way ANOVA, *P < 0.05, **P < 0.01

According to the results obtained from the AD cell model, we examined the expression of the equivalent proteins in the brain tissue of the mouse model. VPA and WT161 inhibited the expression of HDAC1 and HDAC6 in the cerebral cortex and hippocampus of AD mice (Fig. 8). For their effects on the expression of the other three histone deacetylases (SIRT1, SIRT2 and HDAC2), please refer to Additional file 1: Fig. S9. Both VPA and WT161 were able to reduce the expression of p-JNK, JNK3 and c-Jun in the cerebral cortex of AD mice (Fig. 8). The expression of *APP* (Additional file 1: Figure S8) and related secretases was also affected by 18 weeks of treatment of VPA or WT161. The expression of APP β-secretase (BACE1) and γ-secretase (PSEN1) in the cerebral cortex of AD mice was downregulated after drug administration, whereas the expression of α-secretase (ADAM10) was upregulated. The changes in the expression of BACE1 and ADAM10 in the hippocampus were consistent with those in the cerebral cortex, but the expression of PSEN1 was upregulated (Fig. 9). Then, we identified the deposition of Aβ in the brains of mice using thioflavin-S staining and found that VPA and WT161 reduced the size and number of Aβ plaques in the cortex and hippocampus (Fig. 10). Comparable results were obtained from immunohistochemical experiments on brain tissue (Additional file 1: Fig. S9). In addition, Aβ42 in the serum of AD mice was restored to levels similar to those of WT mice (Table 2).

**Table 2:** Detection of plasma Aβ42 levels in various groups of mice by ELISA ($\overline{x }\pm s,n=3$)

Group	Aβ42 concentration (pg/ml)
WT	29.42 ± 5.13**
APP/PS1	68.88 ± 18.56
APP/PS1 + VPA	26.59 ± 5.02**
APP/PS1 + WT161	20.97 ± 4.28**
F	69.391
*P*	0.000

Using the APP/PS1 group as the baseline, one-way ANOVA was used to compare the differences with other treatment groups, **P < 0.01

## Discussion

Alzheimer’s disease is characterized by two classic pathological features: β-amyloid plaque deposition and neurofibrillary tangles of hyperphosphorylated tau. Attempts have been made to treat AD by reducing the synthesis of Aβ, inhibiting the aggregation of Aβ, or removing Aβ deposits. Cleavage of amyloid precursor protein (APP) by β-secretases and γ-secretases yields insoluble Aβ42 product. Due to toxicity and futility, clinical trials for β-secretase inhibitors (Verubecestat [39], Atabecestat [40]) and γ-secretase inhibitors (Semagacestat [41], Avagacestat [42]) were halted [43]. Small molecule inhibitors of RAGE (receptor for advanced glycation end products), such as Azeliragon and FPS-ZM1, may prevent the RAGE-mediated influx of plasma Aβ42 into the brain and lower Aβ42 levels in the brain [44]. RAGE inhibitors have shown a good safety profile in clinical trials, but more research is needed to determine their efficacy [45]. The anti-Aβ monoclonal antibody aducanumab was authorized by the FDA in 2021. Along with the reduced brain amyloid-beta plaques and a slowed decline in cognition in a time- and dose-dependent manner, this new drug is quite expensive, and there are still some doubts about its benefits [46, 47]. In this study, we found that both VPA and WT161 could reduce the expression of APP, β-secretase (BACE1) and γ-secretase (PSEN1), and WT161 could partially restore the expression of α-secretase (ADAM10) in the AD cell model (Fig. 2). This means that both drugs inhibit APP hydrolysis by repressing the expression of β- and γ-secretase along with reducing APP expression. In addition, through a molecular pathway similar to that in the AD cell model, VPA and WT161 also substantially reduced the number and size of Aβ amyloid plaques in the cerebral cortex and hippocampus of APP/PSEN1 mice (Figs. 9 and 10).

VPA and WT161 are HDAC inhibitors, and their effects on AD cell models and AD mice are inseparable from the regulation of HDACs. The four classes of isozymes that make up the HDAC family are class I (HDACs 1–3 and 8), class IIa (HDACs 4, 5, 7 and 9), class IIb (HDACs 6 and 10), class III (sirtuins 1–7) and class IV (HDAC11) [29]. Class I HDACs, particularly HDAC1 and 2, are the most abundant isozymes in brain regions such as the cortex and hippocampus, regulating learning, cognition and memory [48]. HDAC1’s role is not quite clear because it has been linked to both neurotoxic and neuroprotective effects, and the contradictory results still need further investigation [49–51]. The present study found that HDAC1 expression was substantially higher in the cortices of AD mouse models than in wild-type mice (Fig. 8). In the AD cell model, the expression of APP, BACE1, ADAM10, PSEN1 and Aβ42 was altered in accordance with the expression level of HDAC1 (Fig. 3). This means that our results support the neurotoxic effect of HDAC1 to some extent. HDAC6 functions mostly in the cytoplasm and deacetylates nonhistone proteins [52]. HDAC6 overexpression has been observed in the hippocampus and other brain regions of AD patients as well as AD animal models [53–55]. Based on previous studies, HDAC6 may play a role in AD by negatively regulating the expression of GSK-3β (glycogen synthase kinase 3β) and influencing Tau phosphorylation [56–59]. In the current study, we also found that HDAC6 expression was elevated in the AD cell model and in the cortex and hippocampus of AD mice, while VPA and WT161 both drastically reduced HDAC6 expression (Figs. 1 and 8). Moreover, knocking down the expression of HDAC6 in the AD cell model upregulated the expression of ADAM10 while downregulating the expression of BACE1 and PSEN1 (Fig. 3). This indicates that HADC6 knockdown stimulated the nonamyloid-cleaving process of APP in addition to limiting the amyloid-cleaving process of APP, thus restoring Aβ42 to the level of wild-type cells in the AD cell model (Fig. 3). Our results imply that HDAC6 is not only associated with Tau aggregation and stability but is also involved in the regulation of APP secretase expression. We consider HDAC6 to be one of the predominant AD therapeutic targets in the HDAC homologue family.

VPA is a branching short-chain fatty acid that is primarily used to treat seizures and epilepsy. Because of its effect as an HDAC inhibitor, some research has examined its usage as an adjuvant medication in the treatment of cancer, HIV and neurodegenerative diseases [60–62]. VPA was speculated to be a promising agent for the treatment of Alzheimer’s disease more than 10 years ago [63]. The neuroprotective effects and neurogenesis-inducing activities have been described in the following research: Yao et al. showed that VPA might improve memory impairment while lowering Aβ production and senile plaque development [64], Zeng et al. discovered that VPA enhanced neurogenesis via the Wnt pathway and enhanced learning and memory in transgenic mice used as an AD model [65], and Long et al. found a gender difference in VPA-induced neuroprotective effects [66]. In the present study, we found that VPA, a pan-HDAC inhibitor, can inhibit the expression of HDAC1, HDAC2, HDAC6, SIRT1 and SIRT2 in the cortex and hippocampus of AD mice as well as in the AD cell model (Fig. 1, Fig. S2, Fig. 8, Additional file 1: Fig. S9). By inhibiting the expression of HDACs, VPA not only reduced the expression of Aβ42 in the AD cell model but also significantly impaired the deposition of Aβ in the cerebral cortex, hippocampus and entorhinal cortex (Fig. 10, Additional file 1: Fig. S9) and improved the cognitive function of APP/PSEN1 transgenic mice (Figs. 5, 6 and 7). Furthermore, our results indicate that the impact of VPA on the expression of HDAC1 and HDAC2 is dose-dependent. At a concentration of 0.5 mM, VPA inhibits the expression of HDAC6 (Fig. 1C) but has no effect on the expression of HDAC1 and HDAC2, while increasing VPA concentrations to 1 or 2.5 mM could downregulate the expression of HDAC1 and HDAC2 (Fig. 1B and Additional file 1: Fig. S2D). Reinhardt et al. reported that TBX2 can inhibit the transcription of ADAM10, requiring HDAC1 as a co-factor [67], suggesting that the decreased HDAC1 expression can partially relieve the inhibitory effect of TBX2 on ADAM10 expression. Hu et al. found that the application of RNAi to downregulate the expression of HDAC2 resulted in an increasing of the expression level of ADAM10 [68]. We speculate that at higher concentrations, VPA could consequently reduce the expression of ADAM10 via HDAC inhibitions. Previous research has concentrated on the inhibitory effects of VPA on class I and class IIa HDACs, with only one study speculating that VPA could reach the catalytic tunnel of HDAC6 [69]. Our study discovered that VPA could affect HDAC6 expression, but further work is needed to determine whether this was a direct or indirect effect.

In the study conducted by Nau et al., the levels of VPA in both the brain and plasma of mice were measured. The mice were given an intraperitoneal injection of 200 mg/kg of VPA. At 0.25 h after the injection, the concentration of VPA in the mouse brain tissue was 60 μg/g (approximately 0.36 mM) [70]. In the current study, we administered a dosage of 50 mg/kg VPA via intraperitoneal injection for 18 weeks, expecting the brain content of VPA in mice to be approximately 0.09 mM at 0.25 h after each injection, which is one-fourth of the aforementioned study. This concentration is lower than the published IC50 value of VPA (0.4–0.5 mM) [71, 72]. Previous studies have shown that continuous administration at a dose of 30 mg/day for 4 weeks has a positive impact on AD mice [65, 66]. Therefore, we speculate that the cumulative effects of long-term administration are likely the reason why VPA exerts its effects in the brain of AD mice at relatively low concentrations.

To investigate the mechanism of action of HDAC6 inhibition in multiple myeloma, a novel selective HDAC6 inhibitor designated WT161 was developed in 2016 [30]. In follow-up studies, WT161 was found to have adjuvant therapeutic effects against breast cancer [73], retinoblastoma [74] and osteosarcoma [31, 75] and protective properties against arsenic-induced carcinogenesis [76]. Stress-activated JNK [30], EGFR and ERα [73], Bad [74], PTEN and the downstream PI3K/AKT pathway [31, 75] are the targets involved in the regulation of WT161 through inhibition of HDAC6 activity. In the mentioned studies, the acetylation level of α-tubulin was the main criterion for determining the extent of HDAC6 inhibition by WT161, but the effect of WT161 on the HDAC6 expression level was not addressed. As discussed above, HDAC6 is an important AD therapeutic target, and previous research has shown that the selective HDAC6 inhibitors tubastatin A and ACY-1215 could improve cognitive performance in AD mice [76]. In the present study, WT161 was first employed in both cellular and animal models of AD. Hideshima et al. assessed the pharmacokinetic properties of WT161 in mouse, the plasma concentration at 6 h after intravenous injection is approximately 10 ng/ml [30]. However, the ability of WT161 to penetrate the blood–brain barrier remains unknown. We utilized the LC–MS/MS method to quantify the levels of WT161 in the brain tissue of 5 mice. The results indicate that WT161 can indeed penetrate the blood–brain barrier and the average concentration of WT161 in the brain of 5 mice is 6.05 ng/g (approximately 13 nM), which is higher than reported IC50 value of WT161 (0.4 nM) [30]. At a dose of 10 mg/kg/day, WT161 was discovered to be nontoxic (Additional file 1: Fig. S6-S7, Table S1) and substantially reduced Aβ in the cerebral cortex, hippocampus and entorhinal cortex (Fig. 10, Additional file 1: Fig. S9) while enhancing AD mice’s daily behaviour, short-term memory and spatial memory (Figs. 5, 6 and 7).

Previous studies have demonstrated that HDAC6 overexpression activated JNK and enhanced c-Jun phosphorylation [77], while HDAC6 inhibition resulted in a striking reduction in JNK and c-Jun phosphorylation [78, 79]. On the other hand, the expression of BACE1 (β-secretase) and PSEN1 (γ-secretase) can be regulated by the JNK/c-Jun pathway [34–38]. In the current study, we showed that JNK phosphorylation was markedly increased in the cerebral cortex of AD mice (Fig. 8). VPA and WT161 inhibited JNK and c-Jun phosphorylation by repressing the expression of HDAC6 (Fig. 8), further downregulating the expression of BACE1 and PSEN1 (Fig. 9) and ultimately reducing Aβ deposition (Fig. 10, Additional file 1: Fig. S9). Notably, WT161 dramatically increased the expression of ADAM10, a crucial enzyme in the nonamyloidogenic APP processing pathway that cleaves APP to soluble APP-alpha (sAPPa), in the cerebral cortex of AD animals (Fig. 8). However, ADAM10 expression may not be regulated by the JNK pathway [36, 80, 81], so the intermediate link in the regulation of ADAM10 by HDAC6 needs to be further explored.

## Conclusions

We have discovered that VPA and WT161 can inhibit Aβ deposition in vitro and in vivo through the HDAC6-JNK-APP secretase cascade and substantially improve cognitive function in AD mice. VPA has been in clinical use for over 50 years, leading to a relatively comprehensive understanding of its safety and adverse reactions. WT161 is a recently developed HDAC6 inhibitor that has recently been used primarily in tumour therapy trials. We examined the potential value of WT161 for treating AD, initially showing its efficacy and safety in animal models. Overall, our findings reinforce that HDACs are effective therapeutic targets and provide essential preclinical evidence for the clinical evaluation of these two medications for the treatment of Alzheimer’s disease.

### Supplementary Information

**Additional file 1: Fig. S1.** CCK-8 detects drug toxicity in N2a-APPswe. a Cytotoxic effect of VPA on N2a-APPswe. b Cytotoxic effects of WT161 on N2a-APPswe. **Fig. S2.** Effect of VPA and WT161 on the expression of histone deacetylases. a Western blot detection of HDAC2, SIRT1 and SIRT2 expression in N2a-APPswe treated with different concentrations of VPA for 72 h. d Western blot detection of HDAC2, SIRT1 and SIRT2 expression in N2a-APPswe treated with different concentrations of WT161 for 72 h. b-c e-f The results of grayscale scan analysis ($\overline{x }$±s, *n*=3), in which N2a-APPswe treated with VPA and WT161 in group 0 were used as the baseline, and one-way ANOVA was used to compare the differences with other treatment groups, * P < 0.05, ** P < 0.01. **Fig. S3. **Effect of vitamin C on the expression of HDACs and APP metabolism-related proteins. a Western blot detection of HDAC1, APP, ADAM10, BACE1 and PS-1 expression in N2a-APPswe-shHDAC1 cells after 48 h of treatment with different concentration gradients of vitamin C. b-f The results of grayscale scan analysis ($\overline{x }$±s, *n*=3) for N2a-APPswe-shHDAC1 vitamin C treatment group 0 were used as the baseline. g Western blot detection of HDAC1, APP, ADAM10, BACE1 and PS-1 expression in N2a-APPswe-shHDAC6 cells treated with different concentrations of vitamin C for 48 h. h-l The results of grayscale scan analysis ($\overline{x }$±s, *n*=3), in which N2a-APPswe-shHDAC6 vitamin C treatment group 0 was used as the baseline to compare the differences with other treatment groups using one-way ANOVA, *P < 0.05 and **P < 0.01. **Fig. S4. **Effect of the JNK pathway inhibitor SP600125 on APP-related protein expression. a Western blot detection of p-JNK, JNK3, ADAM10, BACE1 and PS-1 expression in N2a-APPswe after 24 h of treatment with SP600125 at concentrations of 5, 10, 20, 40 and 80 μM. b–f The results of grayscale scan analysis ($\overline{x }$±s, *n*=3), in which the N2a- APPswe cell group was the baseline, and the differences with other treatment groups were compared using one-way ANOVA, *P < 0.05 and **P < 0.01. **Fig. S5. **Effect of knockdown of HDAC1 or HDAC6 on the JNK/c-Jun pathway. a Western blot detection of p-JNK, JNK3 and c-Jun expression in N2a-APPswe after stable transfection with shHDAC1 and shHDAC6. b-d The results of grayscale scan analysis ($\overline{x }$±s, *n*=3), in which the N2a-APPswe cell group was used as the baseline for comparison using one-way ANOVA differences with other treatment groups, *P < 0.05, **P < 0.01. **Fig. S6. **Ingestion and weight changes in APP/PS1 mice after VPA and WT161 treatment. a APP/PS1 double transgenic AD mice were treated with VPA and WT161 (*n*=18). b Body weight changes in APP/PS1 double transgenic AD mice after VPA and WT161 treatment using two-way repeated-measures ANOVA to compare the effects of different treatments and times on the feeding and body weight of AD mice. **Fig. S7.** Effect of VPA and WT161 treatment on the organ coefficients of APP/PS1 mice. a-f The organ coefficients of the brain, heart, liver, spleen, lung and kidney in the WT group, APP/PS1 group, VPA group and WT161 group mice (*n*=9). The APP/PS1 group was used as the baseline, and the differences with other treatment groups were compared using one-way ANOVA, *P < 0.05, **P < 0.01. **Fig. S8.** Effects of VPA and WT161 treatment on *APP* expression. qPCR detection of *APP* mRNA expression in the cerebral cortex of each group of mice ($\overline{x }$±s, *n*=4), in which the APP/PS1 group was the baseline, and compared with other treatment groups using one-way ANOVA, ***P < 0.0001. **Fig. S9. **Effects of treatments on the expression of HDACs in the hippocampus and cortex. a Western blot detection of SIRT1, SIRT2 and HDAC2 expression in the cortex of each group of mice. e Western blot detection of SIRT1, SIRT2 and HDAC2 expression in the hippocampus of each group of mice. b-d f-h The results of grayscale scan analysis ($\overline{x }$±s, *n*=3), in which the APP/PS1 group was used as the baseline and one-way ANOVA was used to compare the differences with other treatment groups, *P < 0.05, **P < 0.01. Immunohistochemical detection of VPA and WT161 on brain Aβ amyloid deposition in AD mice. i Immunohistochemistry was performed using mouse-derived 6E10 antibody (1:500) to detect Aβ amyloid deposition in the cortex, hippocampus and internal olfactory cortex of each group of mice (200×, coronal cut). j-k The area ratio of Aβ amyloid plaques in the cortex and hippocampus of each group of mice ($\overline{x }$±s, *n*=6). l-m The number of Aβ amyloid plaques in the cortex and hippocampus of each group of mice ($\overline{x }$±s, *n*=6), all of which were based on the APP/PS1 group. The differences with other treatment groups were compared using one-way ANOVA, *P < 0.05 and **P < 0.01. **Tab. S1.** Serum biochemical indexes of VPA- and WT161-treated APP/PS1 double transgenic AD mice.

## Data Availability

All data generated in this research are available from the corresponding author on reasonable request.

## Abbreviations

AD: Alzheimer’s disease
HDACs: Histone deacetylases
VPA: Valproate
Aβ: Amyloid-β
APP: Amyloid precursor protein
BACE1: β-Site of APP cleaving enzyme
PS-1: Presenilin 1
ADAM10: A disintegrin and metalloprotease 10
MWM: Morris water maze
GLU: Blood glucose
T-CHO: Total cholesterol
TG: Triglycerides
HDL: High-density lipoprotein
LDL: Low-density lipoprotein
AST: Aspartate transaminase
ALT: Alanine transaminase
T-BIL: Total bilirubin
Cr: Creatinine
BUN: Blood urea nitrogen
PFA: Paraformaldehyde
ELISA: Enzyme-linked immunosorbent assay
HR: Horseradish peroxidase
TMB: Tetramethylbenzidine
LC–MS/MS: Liquid chromatography-mass spectrometry/mass spectrometry
