# Supplementary material for: Histone deacetylase inhibitors VPA and WT161 ameliorate the pathological features and cognitive impairments of the APP/PS1 Alzheimer’s disease mouse model by regulating the expression of APP secretases
Source: Alzheimers Res Ther. 2024 Jan 20;16:15. doi: 10.1186/s13195-024-01384-0 (PMC10799458; doi:10.1186/s13195-024-01384-0)
Supplement: Supplementary file 1 — Additional file 1: Fig. S1. CCK-8 detects drug toxicity in N2a-APPswe. a Cytotoxic effect of VPA on N2a-APPswe. b Cytotoxic effects of WT161 on N2a-APPswe. Fig. S2. Effect of VPA and WT161 on the expression of histone deacetylases. a Western blot detection of HDAC2, SIRT1 and SIRT2 expression in N2a-APPswe treated with different concentrations of VPA for 72 h. d Western blot detection of HDAC2, SIRT1 and SIRT2 expression in N2a-APPswe treated with different concentrations of WT161 for 72 h. b-c e-f The results of grayscale scan analysis ($\overline{x }$x¯±s, n=3), in which N2a-APPswe treated with VPA and WT161 in group 0 were used as the baseline, and one-way ANOVA was used to compare the differences with other treatment groups, * P < 0.05, ** P < 0.01. Fig. S3. Effect of vitamin C on the expression of HDACs and APP metabolism-related proteins. a Western blot detection of HDAC1, APP, ADAM10, BACE1 and PS-1 expression in N2a-APPswe-shHDAC1 cells after 48 h of treatment with different concentration gradients of vitamin C. b-f The results of grayscale scan analysis ($\overline{x }$x¯±s, n=3) for N2a-APPswe-shHDAC1 vitamin C treatment group 0 were used as the baseline. g Western blot detection of HDAC1, APP, ADAM10, BACE1 and PS-1 expression in N2a-APPswe-shHDAC6 cells treated with different concentrations of vitamin C for 48 h. h-l The results of grayscale scan analysis ( [file 13195_2024_1384_MOESM1_ESM.zip › Table S1.docx]

**Tab. S1** Serum biochemical indexes of VPA- and WT161-treated APP/PS1 double transgenic AD mice

| Characteristics | WT | APP/PS1 | APP/PS1+VPA | APP/PS1+WT161 | F | *P* |
| --- | --- | --- | --- | --- | --- | --- |
| glucose(mmol/L) | 2.12±1.08 | 2.31±0.56 | 1.48±0.48 | 1.40±0.44 | 1.716 | 0.217 |
| Total cholesterol(mmol/L) | 2.27±0.91 | 1.87±0.03 | 1.66±0.26 | 1.87±0.22 | 1.071 | 0.398 |
| Triglyceride(mmol/L) | 0.40±0.41 | 0.68±0.21 | 0.50±0.45 | 0.37±0.08 | 0.736 | 0.550 |
| High-density lipoprotein cholesterol(mmol/L) | 2.44±0.55 | 1.67±0.27 | 1.55±0.44 | 1.73±0.46 | 3.193 | 0.063 |
| Low-density lipoprotein cholesterol(mmol/L) | 0.16±0.07 | 0.16±0.02 | 0.17±0.03 | 0.10±0.03 | 1.951 | 0.175 |
| Aspartate Transaminase(U/L) | 10.11±1.57** | 14.32±3.58 | 12.87±1.62 | 14.2±2.65 | 9.032 | 0.000 |
| Alanine transaminase(U/L) | 8.02±3.66** | 9.09±3.35 | 9.23±2.34 | 10.29±8.06 | 4.896 | 0.007 |
| Total bilirubin(μmol/L) | 15.17±1.16 | 19.73±2.64 | 17.45±2.99 | 22.01±3.65 | 0.324 | 0.808 |
| Creatinine(μmol/L) | 76.48±7.56 | 80.47±12.96 | 81.9±14.22 | 78.12±12.69 | 0.158 | 0.922 |
| Urea nitrogen(mmol/L) | 6.64±0.79 | 7.97±1.46 | 9.16±2.86 | 10.13±1.28 | 2.881 | 0.080 |

Using the APP/PS1 group as the baseline, one-way ANOVA was used to compare the differences with other treatment groups, **P*< 0.05, ***P* < 0.01.
