# Supplementary figures and images for: Histone deacetylase inhibitors VPA and WT161 ameliorate the pathological features and cognitive impairments of the APP/PS1 Alzheimer’s disease mouse model by regulating the expression of APP secretases
Source: Alzheimers Res Ther. 2024 Jan 20;16:15. doi: 10.1186/s13195-024-01384-0 (PMC10799458; doi:10.1186/s13195-024-01384-0)

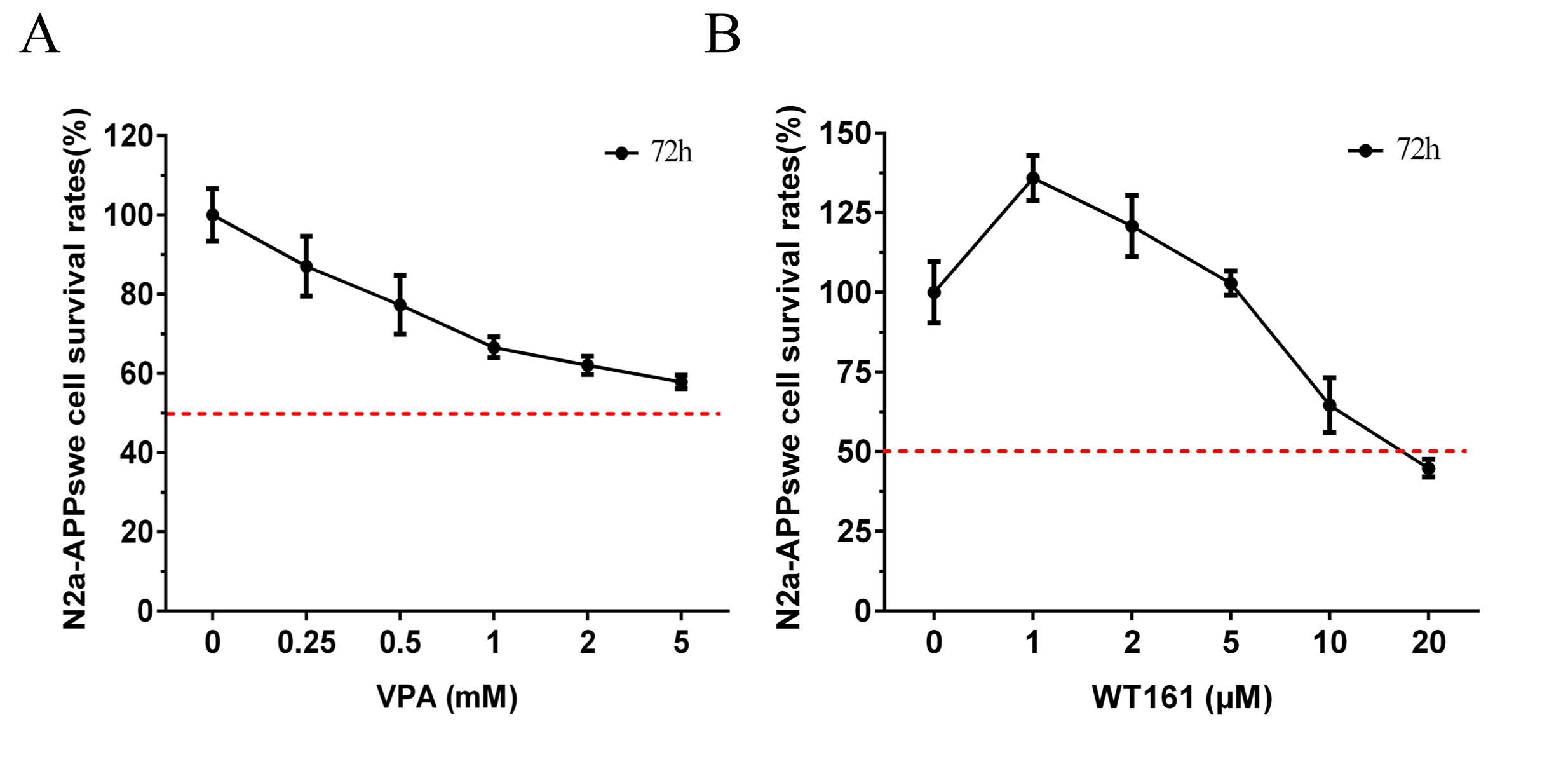

Supplement: Supplementary file 1 — Additional file 1: Fig. S1. CCK-8 detects drug toxicity in N2a-APPswe. a Cytotoxic effect of VPA on N2a-APPswe. b Cytotoxic effects of WT161 on N2a-APPswe. Fig. S2. Effect of VPA and WT161 on the expression of histone deacetylases. a Western blot detection of HDAC2, SIRT1 and SIRT2 expression in N2a-APPswe treated with different concentrations of VPA for 72 h. d Western blot detection of HDAC2, SIRT1 and SIRT2 expression in N2a-APPswe treated with different concentrations of WT161 for 72 h. b-c e-f The results of grayscale scan analysis ($\overline{x }$x¯±s, n=3), in which N2a-APPswe treated with VPA and WT161 in group 0 were used as the baseline, and one-way ANOVA was used to compare the differences with other treatment groups, * P < 0.05, ** P < 0.01. Fig. S3. Effect of vitamin C on the expression of HDACs and APP metabolism-related proteins. a Western blot detection of HDAC1, APP, ADAM10, BACE1 and PS-1 expression in N2a-APPswe-shHDAC1 cells after 48 h of treatment with different concentration gradients of vitamin C. b-f The results of grayscale scan analysis ($\overline{x }$x¯±s, n=3) for N2a-APPswe-shHDAC1 vitamin C treatment group 0 were used as the baseline. g Western blot detection of HDAC1, APP, ADAM10, BACE1 and PS-1 expression in N2a-APPswe-shHDAC6 cells treated with different concentrations of vitamin C for 48 h. h-l The results of grayscale scan analysis ( [file 13195_2024_1384_MOESM1_ESM.zip › Figure S1.tif]

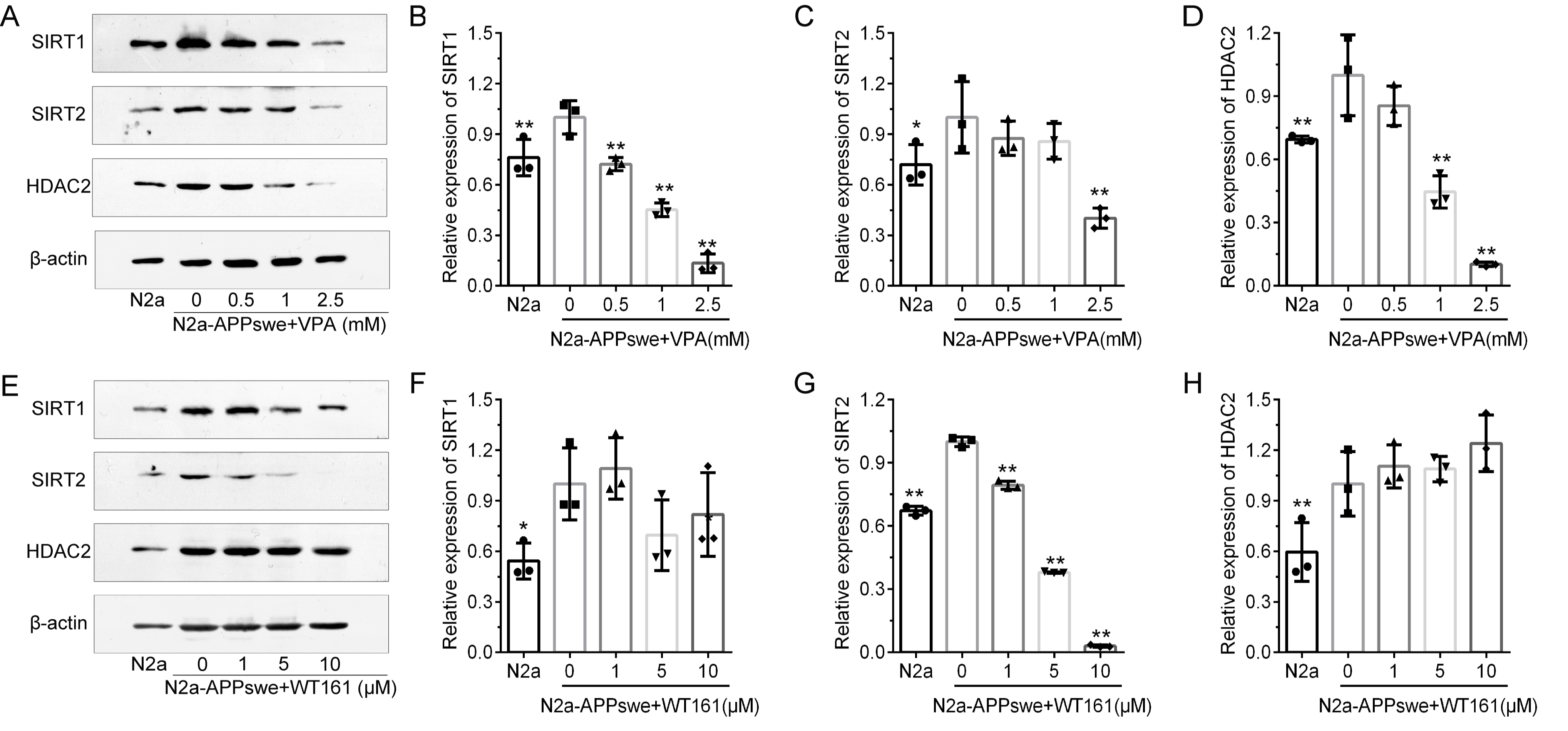

Supplement: Supplementary file 1 — Additional file 1: Fig. S1. CCK-8 detects drug toxicity in N2a-APPswe. a Cytotoxic effect of VPA on N2a-APPswe. b Cytotoxic effects of WT161 on N2a-APPswe. Fig. S2. Effect of VPA and WT161 on the expression of histone deacetylases. a Western blot detection of HDAC2, SIRT1 and SIRT2 expression in N2a-APPswe treated with different concentrations of VPA for 72 h. d Western blot detection of HDAC2, SIRT1 and SIRT2 expression in N2a-APPswe treated with different concentrations of WT161 for 72 h. b-c e-f The results of grayscale scan analysis ($\overline{x }$x¯±s, n=3), in which N2a-APPswe treated with VPA and WT161 in group 0 were used as the baseline, and one-way ANOVA was used to compare the differences with other treatment groups, * P < 0.05, ** P < 0.01. Fig. S3. Effect of vitamin C on the expression of HDACs and APP metabolism-related proteins. a Western blot detection of HDAC1, APP, ADAM10, BACE1 and PS-1 expression in N2a-APPswe-shHDAC1 cells after 48 h of treatment with different concentration gradients of vitamin C. b-f The results of grayscale scan analysis ($\overline{x }$x¯±s, n=3) for N2a-APPswe-shHDAC1 vitamin C treatment group 0 were used as the baseline. g Western blot detection of HDAC1, APP, ADAM10, BACE1 and PS-1 expression in N2a-APPswe-shHDAC6 cells treated with different concentrations of vitamin C for 48 h. h-l The results of grayscale scan analysis ( [file 13195_2024_1384_MOESM1_ESM.zip › Figure S2.tif]

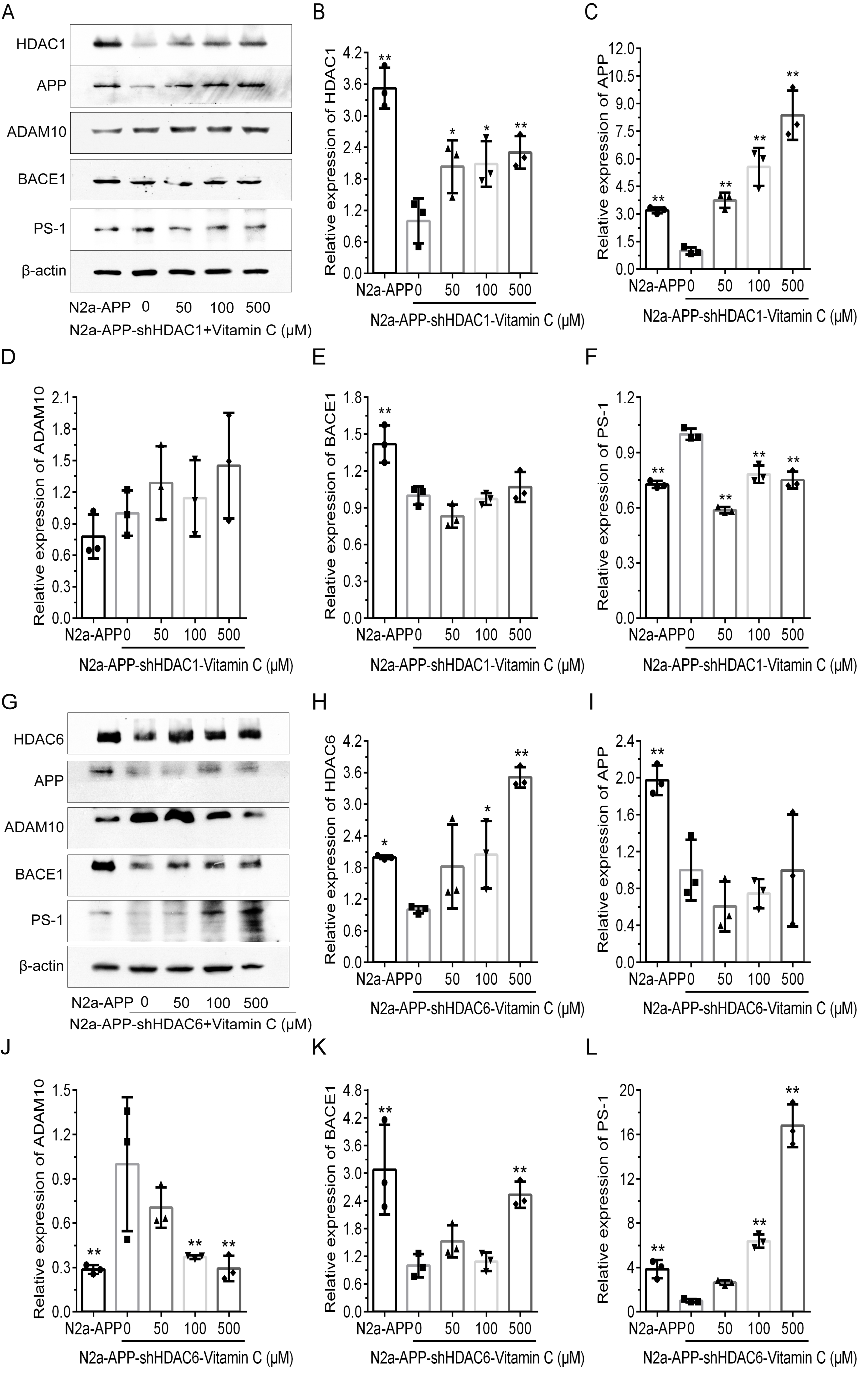

Supplement: Supplementary file 1 — Additional file 1: Fig. S1. CCK-8 detects drug toxicity in N2a-APPswe. a Cytotoxic effect of VPA on N2a-APPswe. b Cytotoxic effects of WT161 on N2a-APPswe. Fig. S2. Effect of VPA and WT161 on the expression of histone deacetylases. a Western blot detection of HDAC2, SIRT1 and SIRT2 expression in N2a-APPswe treated with different concentrations of VPA for 72 h. d Western blot detection of HDAC2, SIRT1 and SIRT2 expression in N2a-APPswe treated with different concentrations of WT161 for 72 h. b-c e-f The results of grayscale scan analysis ($\overline{x }$x¯±s, n=3), in which N2a-APPswe treated with VPA and WT161 in group 0 were used as the baseline, and one-way ANOVA was used to compare the differences with other treatment groups, * P < 0.05, ** P < 0.01. Fig. S3. Effect of vitamin C on the expression of HDACs and APP metabolism-related proteins. a Western blot detection of HDAC1, APP, ADAM10, BACE1 and PS-1 expression in N2a-APPswe-shHDAC1 cells after 48 h of treatment with different concentration gradients of vitamin C. b-f The results of grayscale scan analysis ($\overline{x }$x¯±s, n=3) for N2a-APPswe-shHDAC1 vitamin C treatment group 0 were used as the baseline. g Western blot detection of HDAC1, APP, ADAM10, BACE1 and PS-1 expression in N2a-APPswe-shHDAC6 cells treated with different concentrations of vitamin C for 48 h. h-l The results of grayscale scan analysis ( [file 13195_2024_1384_MOESM1_ESM.zip › Figure S3.tif]

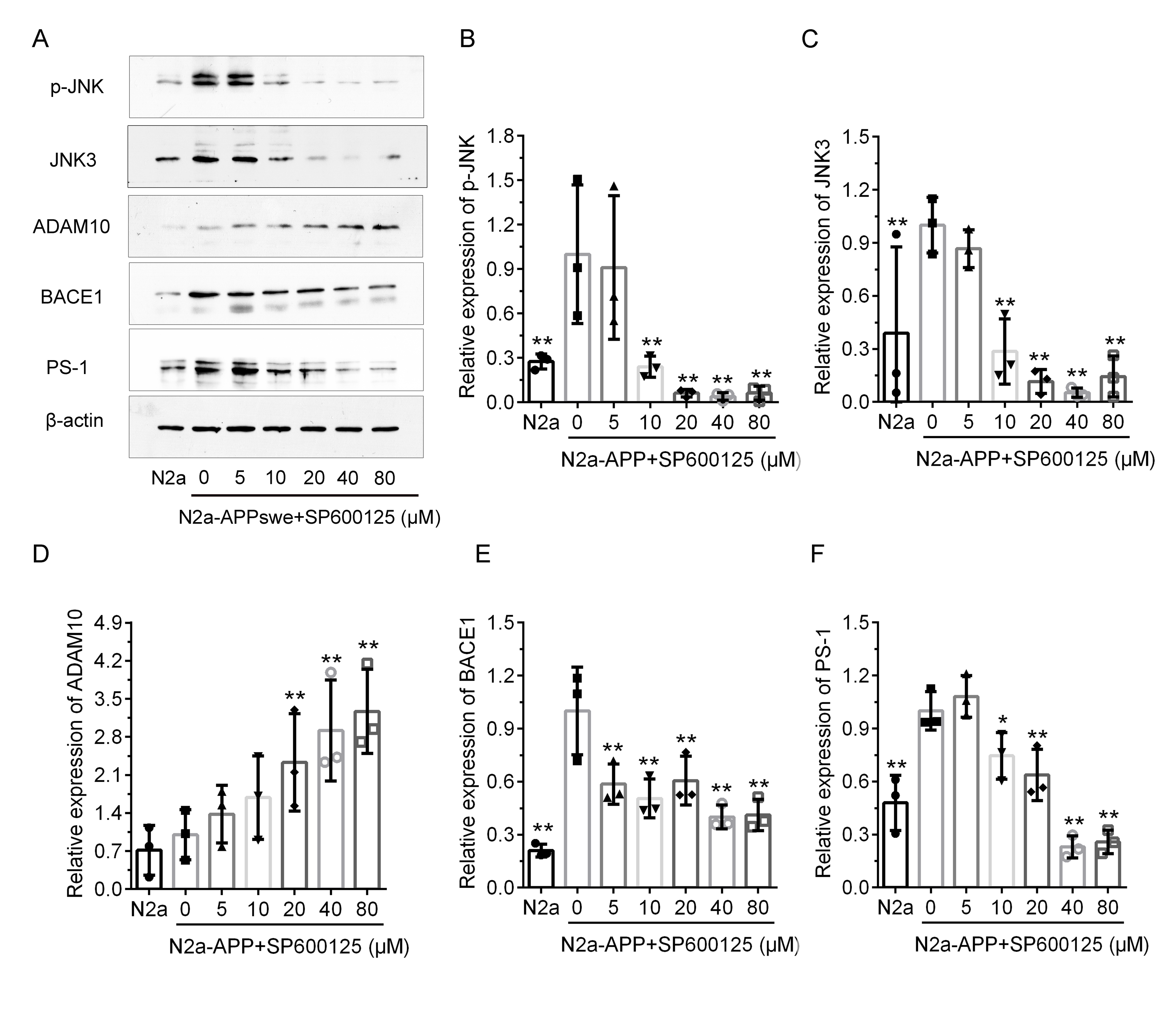

Supplement: Supplementary file 1 — Additional file 1: Fig. S1. CCK-8 detects drug toxicity in N2a-APPswe. a Cytotoxic effect of VPA on N2a-APPswe. b Cytotoxic effects of WT161 on N2a-APPswe. Fig. S2. Effect of VPA and WT161 on the expression of histone deacetylases. a Western blot detection of HDAC2, SIRT1 and SIRT2 expression in N2a-APPswe treated with different concentrations of VPA for 72 h. d Western blot detection of HDAC2, SIRT1 and SIRT2 expression in N2a-APPswe treated with different concentrations of WT161 for 72 h. b-c e-f The results of grayscale scan analysis ($\overline{x }$x¯±s, n=3), in which N2a-APPswe treated with VPA and WT161 in group 0 were used as the baseline, and one-way ANOVA was used to compare the differences with other treatment groups, * P < 0.05, ** P < 0.01. Fig. S3. Effect of vitamin C on the expression of HDACs and APP metabolism-related proteins. a Western blot detection of HDAC1, APP, ADAM10, BACE1 and PS-1 expression in N2a-APPswe-shHDAC1 cells after 48 h of treatment with different concentration gradients of vitamin C. b-f The results of grayscale scan analysis ($\overline{x }$x¯±s, n=3) for N2a-APPswe-shHDAC1 vitamin C treatment group 0 were used as the baseline. g Western blot detection of HDAC1, APP, ADAM10, BACE1 and PS-1 expression in N2a-APPswe-shHDAC6 cells treated with different concentrations of vitamin C for 48 h. h-l The results of grayscale scan analysis ( [file 13195_2024_1384_MOESM1_ESM.zip › Figure S4.tif]

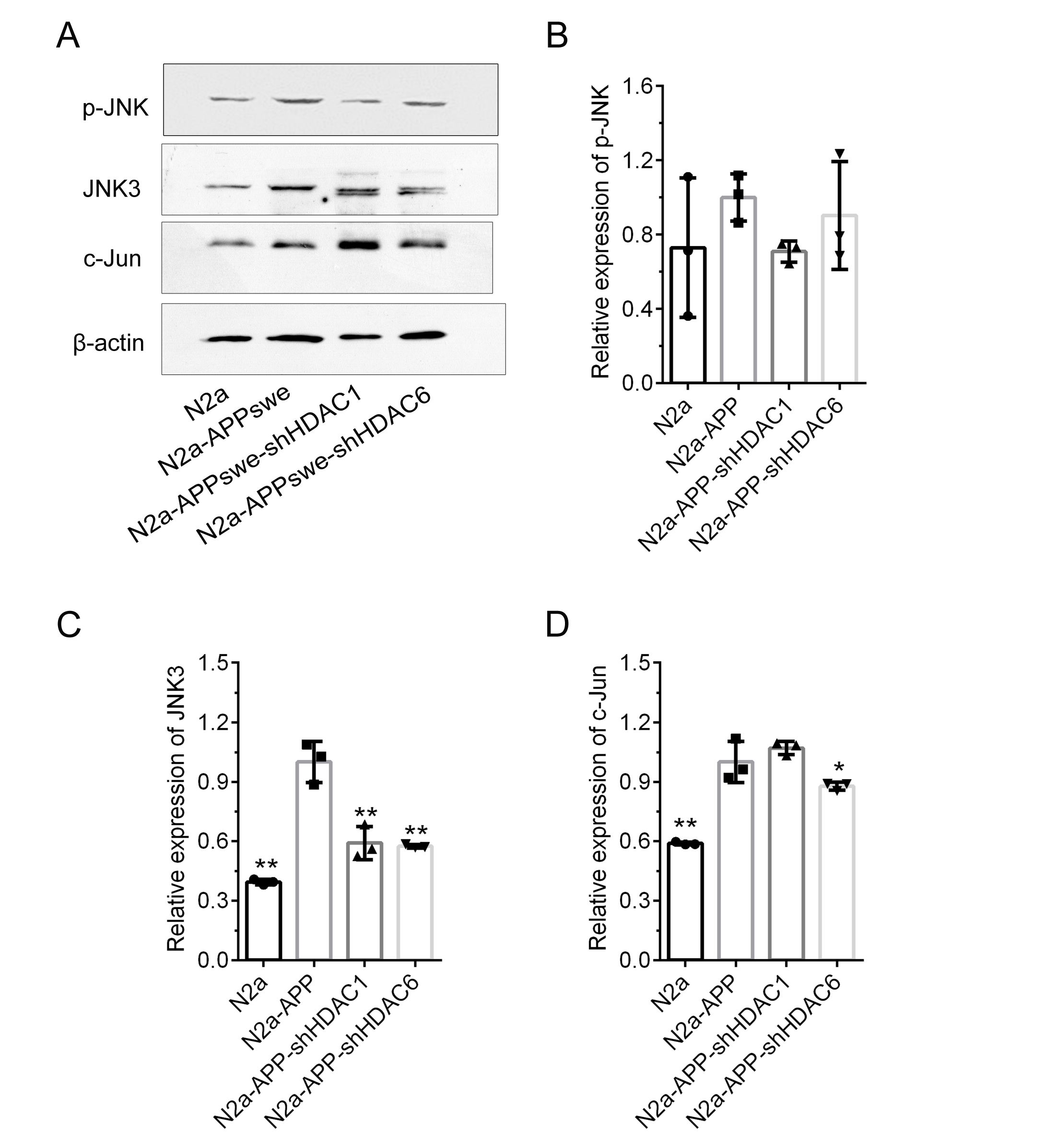

Supplement: Supplementary file 1 — Additional file 1: Fig. S1. CCK-8 detects drug toxicity in N2a-APPswe. a Cytotoxic effect of VPA on N2a-APPswe. b Cytotoxic effects of WT161 on N2a-APPswe. Fig. S2. Effect of VPA and WT161 on the expression of histone deacetylases. a Western blot detection of HDAC2, SIRT1 and SIRT2 expression in N2a-APPswe treated with different concentrations of VPA for 72 h. d Western blot detection of HDAC2, SIRT1 and SIRT2 expression in N2a-APPswe treated with different concentrations of WT161 for 72 h. b-c e-f The results of grayscale scan analysis ($\overline{x }$x¯±s, n=3), in which N2a-APPswe treated with VPA and WT161 in group 0 were used as the baseline, and one-way ANOVA was used to compare the differences with other treatment groups, * P < 0.05, ** P < 0.01. Fig. S3. Effect of vitamin C on the expression of HDACs and APP metabolism-related proteins. a Western blot detection of HDAC1, APP, ADAM10, BACE1 and PS-1 expression in N2a-APPswe-shHDAC1 cells after 48 h of treatment with different concentration gradients of vitamin C. b-f The results of grayscale scan analysis ($\overline{x }$x¯±s, n=3) for N2a-APPswe-shHDAC1 vitamin C treatment group 0 were used as the baseline. g Western blot detection of HDAC1, APP, ADAM10, BACE1 and PS-1 expression in N2a-APPswe-shHDAC6 cells treated with different concentrations of vitamin C for 48 h. h-l The results of grayscale scan analysis ( [file 13195_2024_1384_MOESM1_ESM.zip › Figure S5.tif]

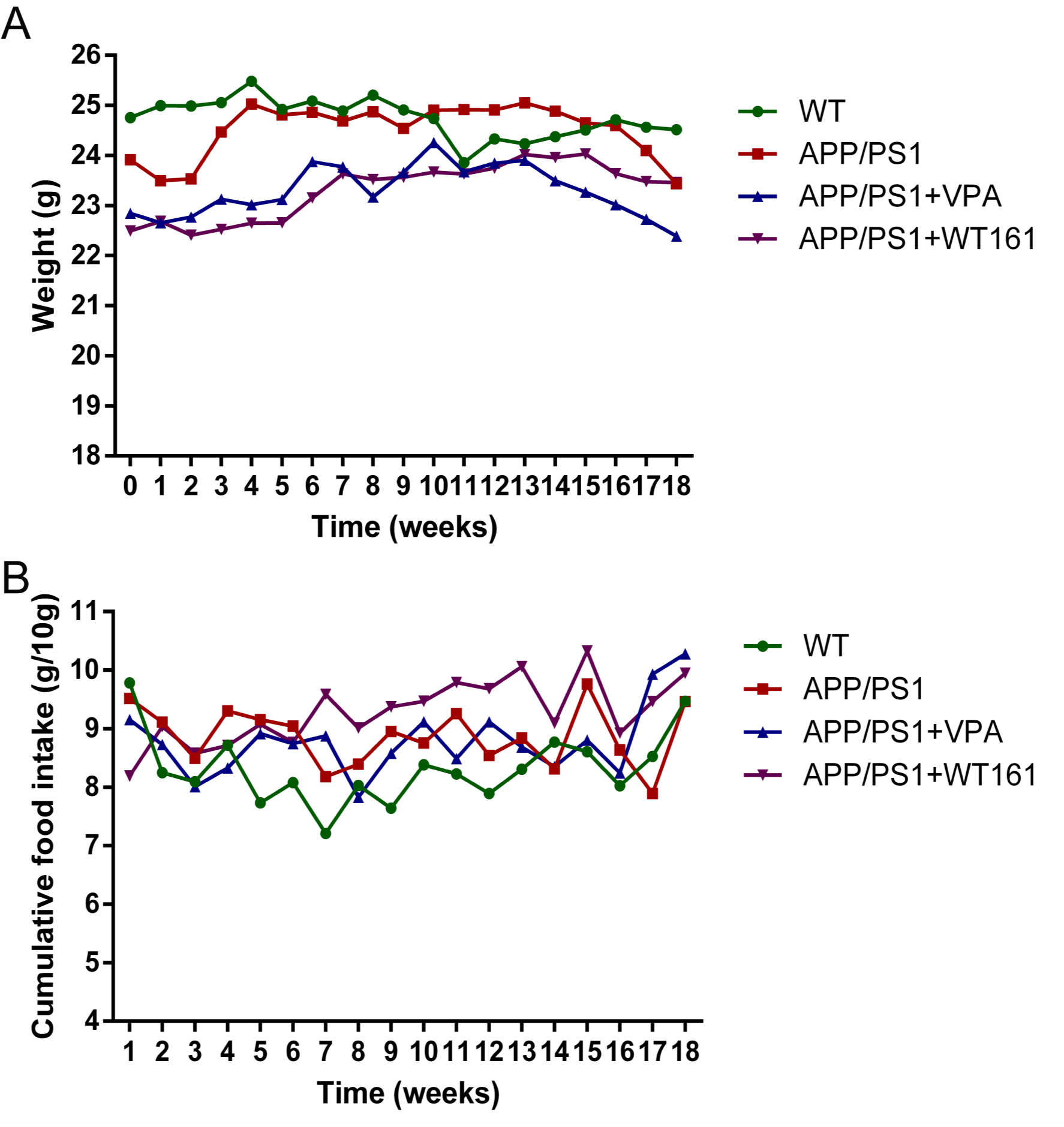

Supplement: Supplementary file 1 — Additional file 1: Fig. S1. CCK-8 detects drug toxicity in N2a-APPswe. a Cytotoxic effect of VPA on N2a-APPswe. b Cytotoxic effects of WT161 on N2a-APPswe. Fig. S2. Effect of VPA and WT161 on the expression of histone deacetylases. a Western blot detection of HDAC2, SIRT1 and SIRT2 expression in N2a-APPswe treated with different concentrations of VPA for 72 h. d Western blot detection of HDAC2, SIRT1 and SIRT2 expression in N2a-APPswe treated with different concentrations of WT161 for 72 h. b-c e-f The results of grayscale scan analysis ($\overline{x }$x¯±s, n=3), in which N2a-APPswe treated with VPA and WT161 in group 0 were used as the baseline, and one-way ANOVA was used to compare the differences with other treatment groups, * P < 0.05, ** P < 0.01. Fig. S3. Effect of vitamin C on the expression of HDACs and APP metabolism-related proteins. a Western blot detection of HDAC1, APP, ADAM10, BACE1 and PS-1 expression in N2a-APPswe-shHDAC1 cells after 48 h of treatment with different concentration gradients of vitamin C. b-f The results of grayscale scan analysis ($\overline{x }$x¯±s, n=3) for N2a-APPswe-shHDAC1 vitamin C treatment group 0 were used as the baseline. g Western blot detection of HDAC1, APP, ADAM10, BACE1 and PS-1 expression in N2a-APPswe-shHDAC6 cells treated with different concentrations of vitamin C for 48 h. h-l The results of grayscale scan analysis ( [file 13195_2024_1384_MOESM1_ESM.zip › Figure S6.tif]

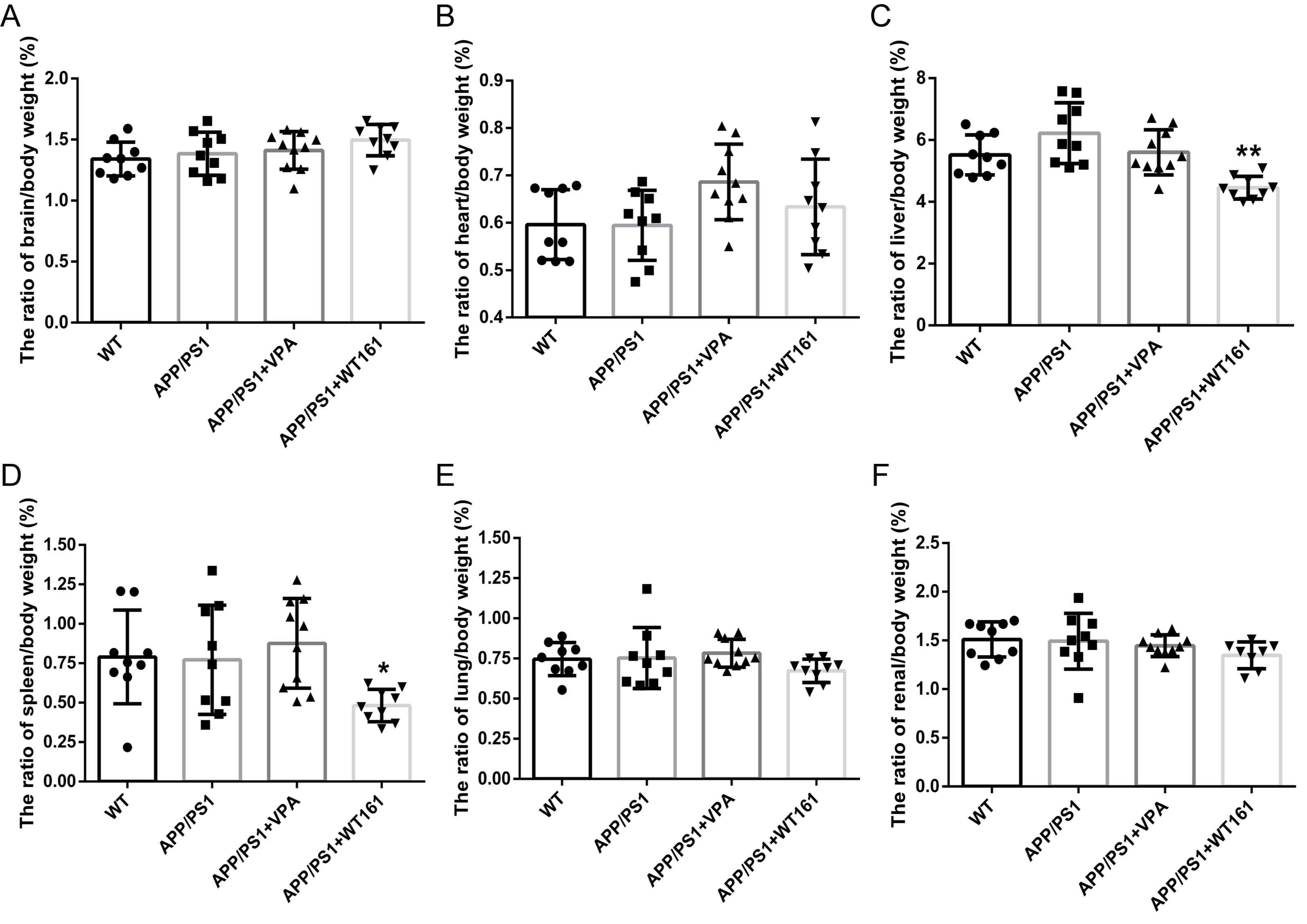

Supplement: Supplementary file 1 — Additional file 1: Fig. S1. CCK-8 detects drug toxicity in N2a-APPswe. a Cytotoxic effect of VPA on N2a-APPswe. b Cytotoxic effects of WT161 on N2a-APPswe. Fig. S2. Effect of VPA and WT161 on the expression of histone deacetylases. a Western blot detection of HDAC2, SIRT1 and SIRT2 expression in N2a-APPswe treated with different concentrations of VPA for 72 h. d Western blot detection of HDAC2, SIRT1 and SIRT2 expression in N2a-APPswe treated with different concentrations of WT161 for 72 h. b-c e-f The results of grayscale scan analysis ($\overline{x }$x¯±s, n=3), in which N2a-APPswe treated with VPA and WT161 in group 0 were used as the baseline, and one-way ANOVA was used to compare the differences with other treatment groups, * P < 0.05, ** P < 0.01. Fig. S3. Effect of vitamin C on the expression of HDACs and APP metabolism-related proteins. a Western blot detection of HDAC1, APP, ADAM10, BACE1 and PS-1 expression in N2a-APPswe-shHDAC1 cells after 48 h of treatment with different concentration gradients of vitamin C. b-f The results of grayscale scan analysis ($\overline{x }$x¯±s, n=3) for N2a-APPswe-shHDAC1 vitamin C treatment group 0 were used as the baseline. g Western blot detection of HDAC1, APP, ADAM10, BACE1 and PS-1 expression in N2a-APPswe-shHDAC6 cells treated with different concentrations of vitamin C for 48 h. h-l The results of grayscale scan analysis ( [file 13195_2024_1384_MOESM1_ESM.zip › Figure S7.tif]

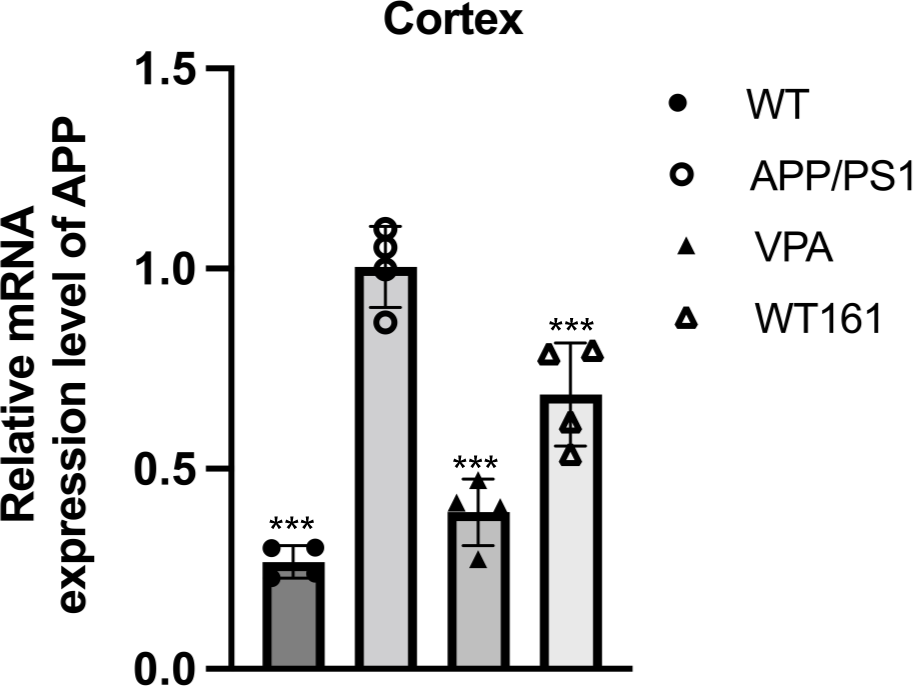

Supplement: Supplementary file 1 — Additional file 1: Fig. S1. CCK-8 detects drug toxicity in N2a-APPswe. a Cytotoxic effect of VPA on N2a-APPswe. b Cytotoxic effects of WT161 on N2a-APPswe. Fig. S2. Effect of VPA and WT161 on the expression of histone deacetylases. a Western blot detection of HDAC2, SIRT1 and SIRT2 expression in N2a-APPswe treated with different concentrations of VPA for 72 h. d Western blot detection of HDAC2, SIRT1 and SIRT2 expression in N2a-APPswe treated with different concentrations of WT161 for 72 h. b-c e-f The results of grayscale scan analysis ($\overline{x }$x¯±s, n=3), in which N2a-APPswe treated with VPA and WT161 in group 0 were used as the baseline, and one-way ANOVA was used to compare the differences with other treatment groups, * P < 0.05, ** P < 0.01. Fig. S3. Effect of vitamin C on the expression of HDACs and APP metabolism-related proteins. a Western blot detection of HDAC1, APP, ADAM10, BACE1 and PS-1 expression in N2a-APPswe-shHDAC1 cells after 48 h of treatment with different concentration gradients of vitamin C. b-f The results of grayscale scan analysis ($\overline{x }$x¯±s, n=3) for N2a-APPswe-shHDAC1 vitamin C treatment group 0 were used as the baseline. g Western blot detection of HDAC1, APP, ADAM10, BACE1 and PS-1 expression in N2a-APPswe-shHDAC6 cells treated with different concentrations of vitamin C for 48 h. h-l The results of grayscale scan analysis ( [file 13195_2024_1384_MOESM1_ESM.zip › Figure S8.tif]

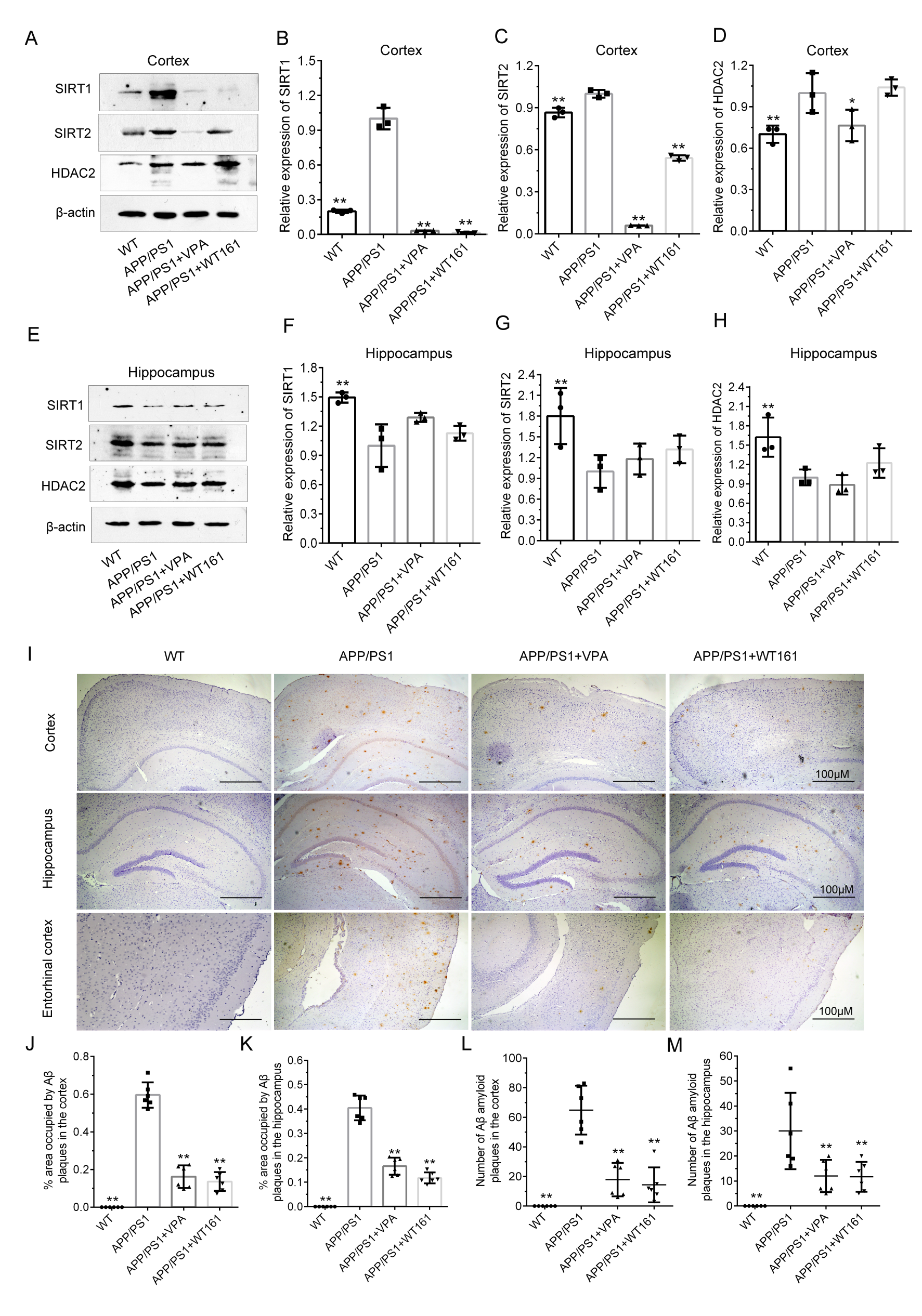

Supplement: Supplementary file 1 — Additional file 1: Fig. S1. CCK-8 detects drug toxicity in N2a-APPswe. a Cytotoxic effect of VPA on N2a-APPswe. b Cytotoxic effects of WT161 on N2a-APPswe. Fig. S2. Effect of VPA and WT161 on the expression of histone deacetylases. a Western blot detection of HDAC2, SIRT1 and SIRT2 expression in N2a-APPswe treated with different concentrations of VPA for 72 h. d Western blot detection of HDAC2, SIRT1 and SIRT2 expression in N2a-APPswe treated with different concentrations of WT161 for 72 h. b-c e-f The results of grayscale scan analysis ($\overline{x }$x¯±s, n=3), in which N2a-APPswe treated with VPA and WT161 in group 0 were used as the baseline, and one-way ANOVA was used to compare the differences with other treatment groups, * P < 0.05, ** P < 0.01. Fig. S3. Effect of vitamin C on the expression of HDACs and APP metabolism-related proteins. a Western blot detection of HDAC1, APP, ADAM10, BACE1 and PS-1 expression in N2a-APPswe-shHDAC1 cells after 48 h of treatment with different concentration gradients of vitamin C. b-f The results of grayscale scan analysis ($\overline{x }$x¯±s, n=3) for N2a-APPswe-shHDAC1 vitamin C treatment group 0 were used as the baseline. g Western blot detection of HDAC1, APP, ADAM10, BACE1 and PS-1 expression in N2a-APPswe-shHDAC6 cells treated with different concentrations of vitamin C for 48 h. h-l The results of grayscale scan analysis ( [file 13195_2024_1384_MOESM1_ESM.zip › Figure S9.tif]
